# In Vitro Techniques for Microleakage Evaluation of Coronary Restorative Materials: A Scoping and Mapping Review

**DOI:** 10.3390/jfb16060210

**Published:** 2025-06-04

**Authors:** Sofia Vieira, Carlos Miguel Marto, Ana Coelho, Inês Amaro, Inês Francisco, Francisco Vale, Siri Paulo, Manuel Marques Ferreira, Eunice Carrilho, Anabela Paula

**Affiliations:** 1 Faculty of Medicine, Institute of Integrated Clinical Practice, University of Coimbra, 3000-075 Coimbra, Portugal; sofia_vieira2000@hotmail.com (S.V.); cmiguel.marto@uc.pt (C.M.M.); anasofiacoelho@gmail.com (A.C.); ines.amaros@hotmail.com (I.A.);; 2 Faculty of Medicine, Institute of Experimental Pathology, University of Coimbra, 3000-548 Coimbra, Portugal; 3 Laboratory of Evidence-Based and Precision Dentistry, Faculty of Medicine, University of Coimbra, 3000-075 Coimbra, Portugal; ines70.francisco@gmail.com (I.F.); fvale@fmed.uc.pt (F.V.); m.mferreira@netcabo.pt (M.M.F.); 4 Faculty of Medicine, Genetics and Oncobiology (CIMAGO), Area of Environment, Coimbra Institute for Clinical and Biomedical Research (iCBR), University of Coimbra, 3000-548 Coimbra, Portugal; 5 Centre for Innovate Biomedicine and Biotechnology (CIBB), University of Coimbra, 3000-548 Coimbra, Portugal; 6 Clinical Academic Center of Coimbra (CACC), University of Coimbra, 3000-548 Coimbra, Portugal; 7 Faculty of Medicine, Institute of Biophysics, University of Coimbra, 3000-548 Coimbra, Portugal; 8 Centre for Mechanical Engineering, Materials and Processes (CEMMPRE), Advanced Production and Intelligent Systems (ARISE), Polo III, University of Coimbra, 3030-788 Coimbra, Portugal; 9 Faculty of Medicine, Institute of Orthodontics, University of Coimbra, 3000-075 Coimbra, Portugal; 10 Faculty of Medicine, Institute of Endodontics, University of Coimbra, 3000-075 Coimbra, Portugal; sirivpaulo@gmail.com; 11 Avenida Bissaya Barreto, Blocos de Celas, 3000-075 Coimbra, Portugal

**Keywords:** microleakage, restorative dentistry, scoping review, in vitro assessment

## Abstract

Objectives: To identify the in vitro techniques used for a microleakage evaluation of restorative materials of coronary structure through a scoping and mapping review. Data: This scoping review aims to answer the following question: “what methods are currently for the microleakage assessment of coronary restorative materials?” Sources: The Medline, Embase, Cochrane Library, and Web of Science databases were searched to identify relevant studies published between 2020 and 2024. The results obtained were grouped according to the evaluation method, and a narrative synthesis was made. Study Selection: The literature search identified 1014 articles, and 353 publications were excluded by title and abstract screening. From the remaining 297, thirty-three were excluded. Additionally, 8 articles could not be retrieved, which resulted in the inclusion of 256 articles. The results were grouped according to the type of microleakage evaluation method, resulting in four major categories: colorimetric, imaging, nuclear medicine, and microbiological methods. Conclusions: Currently, several microleakage assessment techniques are being used, with the dye method being the most reported one. Several variations in the experimental protocols exist, which make it difficult to compare the results. The use of dyes and nuclear medicine methods is sensitive and can be used to evaluate microleakage.

## 1. Introduction

The development of dental adhesion technology and the increasing demands for more durable and predictive restorative treatments have led to advances in restorative dentistry, including the development of a wide range of materials for direct and indirect restorative procedures with different indications, technique sensitivity, and limitations [1]. However, the clinical longevity of dental restorations is still a current concern, mainly due to the degradation of the adhesive interface over time, which allows the phenomenon of microleakage [1,2,3]. Kidd in 1976 defined microleakage as the passage of microorganisms and their toxins, ionic particles, molecules, and oral fluids between the cavity wall and the applied restorative material, resulting in long-term repercussions for the restored tooth, namely marginal discoloration, secondary caries, subsequent loss of retention, postoperative sensitivity, and pathological pulp alterations [1,2,3,4,5]. Microleakage outcomes vary depending on the material properties, bonding strategy and quality, and the substrate nature [4]. For instance, the lack of adhesive properties associated with dental amalgam, as well as the thermal expansion coefficient different from the dental substrate, favors the formation of interfacial gaps [6,7,8]. However, all the materials used in direct and indirect restoration techniques have inherent limitations that can lead to microleakage [1].

The market presents a wide range of composite resins for several clinical indications. However, their therapeutic success is highly sensitive to the technique, depending on factors such as the material dimensional changes due to polymerization shrinkage (i.e., the contraction forces originated inside the material that are transmitted, in part, to the adhesive interface), which results in cusp deflexion and gap formation [2,3,9,10]. The cavity shape also influences the restorative material adaptation to the margins, with higher C-factor values resulting in greater polymerization shrinkage [2,10,11]. Also, since enamel and dentin are tissues with different characteristics (with dentin presenting a more heterogenous and hydrophilic nature), the bonding strategy to different dental tissues must be adapted, avoiding situations such as over-etched, over-dried, or over-wetted dentin that culminates in a weak bond and the degradation of the restorative interface [1,2]. In addition, depending on the restorative material used, the larger coefficient of thermal expansion and the non-matching moduli of elasticity with the dental tissues also contribute to the failure of a mechanical bond, leading to microleakage [10]. In indirect restoration techniques, the improper marginal fit, as well as the subgingival location of the cavity margins, may lead to cement dissolution culminating in microleakage [12,13].

Thus, although adhesion is a well-established and predictable clinical procedure, there is a need to improve materials and techniques to minimize marginal microleakage and increase the predictability and durability of treatments [1,2,3]. Hence, microleakage studies are of paramount importance. These studies can be performed clinically or using in vitro models, allowing for the evaluation of the materials’ performance, combination of materials, and restorative techniques [1,2,3,4]. Despite that the obtained results cannot be directly extrapolated to the clinic, the in vitro microleakage assessment is the most used model and allows us to understand the physical and chemical phenomena suffered by restorative materials after being placed in cavities and predict their clinical behavior [12,14,15]. Many techniques and methodologies have been reported in the literature to test microleakage in vitro, which can be subclassified as qualitative or quantitative [4]. When performing such techniques, the oral environment can be simulated by water storage and thermocycling of the samples [4,10,16]. Dye penetration techniques are the most employed due to their low cost, easy methodology, and technical and equipment requirements [5,17,18]. However, they present some limitations, namely the destruction of the samples and a limited number of sections for assessment, which can lead to leakage underestimation. In addition, variations in dentin permeability can bias the results [5,17,18]. Microbiological methods use cariogenic bacteria, which may mimic oral conditions more accurately and do not destroy the samples. Nevertheless, they require a long period of experimentation and only evaluate the bacterial passage, disregarding the bacterial metabolites, fluids, toxins, or ions [19]. The nuclear medicine methods, evaluating radioisotope penetration, are non-destructive and highly sensitive. However, due to the small size of the isotopes, they can pass through gaps in tooth structure and result in misinterpretation of the microleakage [20,21,22,23]. Furthermore, radioisotopes such as Ca45 have an affinity for restorative materials and tooth structure, which may lead to measurement errors [22]. The imaging techniques allow for visualization and the two-dimensional (2D) or three-dimensional (3D) evaluation of the restoration interface, using or not to organic or fluorescent dyes or chemical markers [5]. Micro-CT is a non-destructive method that allows for the 3D reconstruction of the samples and examines internal aspects such as the restorative interface, irrespective of a sample’s shape or dimensions. However, materials without sufficient radiopacity and with low or no filler content, such as some dental adhesives, are difficult to visualize through software reconstruction and, consequently, the discrimination between adhesive and air is challenging [24].

In this sense, none of the tests can be considered a gold standard, and their results tend to present high variability, due to the different testing protocols [4,14]. Thus, evaluating the methods’ reliability is strongly recommended, and it is necessary to standardize and define clear protocols to assess microleakage in order to obtain accurate results and their comparison [4].

Thus, the present study aims to identify the in vitro techniques used for a microleakage evaluation of restorative materials of the coronary structure through a scoping and mapping review.

## 2. Materials and Methods

The present review was planned and reported following the Preferred Reporting Items for Systematic Reviews and Meta-Analysis, Scoping Reviews extension (PRISMA-ScR) guidelines, and the methodology framework proposed by Arksey and O’Malley [25,26]. The PRISMA-ScR checklist is presented as Supplementary File Table S1.

Review questions

This scoping review aims to answer the following question: “what methods are currently for the microleakage assessment of coronary restorative materials?” The formulation of this question followed the population/participants, concept, and context (PCC) strategy, with the population being the teeth in need of restorative procedures, the concept the microleakage assessment techniques, and the context the coronary restorations using definitive or temporary materials [27]. In addition to the main question, this review also aims to answer three secondary questions: (1) What are the most used methods for microleakage evaluation? (2) What protocols are being used for each method? (3) What is the origin of the samples?

Protocol and registration

The review protocol was registered in the Open Science Framework (OSF) database and is available at https://osf.io/j493m/ (accessed on 10 May 2024), with the following DOI 10.17605/OSF.IO/J493M.

Search strategy and information sources

A literature search was performed in Medline (through PubMed), Embase, Web of Science (all databases), and Cochrane Library databases. A search formula combining MeSH terms and keywords was created for PubMed and adapted for the other databases. The latest search was performed on the 18th of March 2024, and a filter of publication date was applied to restrict the search results to the time between 2020 and 2024 to obtain the most updated results on the microleakage assessment techniques being currently used. The database search strategies and filters used are presented in Supplementary Table S2. The reference list of potentially included articles was searched for additional relevant studies.

Eligibility Criteria and Study Selection

The results were imported into Rayyan software (Qatar Computing Research Institute), and duplicates were automatically removed. Next, studies were screened based on title and abstract. Later, the full texts were retrieved, and articles were selected for inclusion.

The selection was based on pre-defined inclusion and exclusion criteria, which are presented in Table 1. The selection process was performed independently by two authors. A third author was consulted when necessary, and a decision was reached by consensus.

Data Extraction Process

From all included articles and to map the existing information on this topic, the following information was collected: type of microleakage assessment technique used and methods, geographic region and country of the publication, publication language, authors, publication type (journal articles, book chapter, or thesis) and journal where the article was published.

Considering the number of studies obtained, a sampling methodology was used, and the five most recent articles for each microleakage evaluation method were selected for detailed data extraction [28]. The following data were extracted: author(s) and publication year, sample characterization (teeth type, experimental and control groups, number of samples, and restoration type), the protocol used and respective variables (technique applied, exposition conditions, products concentrations, temperature, setting images, magnification used, and scoring systems) and assessment type (quantitative, qualitative, and semi-quantitative).

Synthesis of results

The results were grouped according to the type of microleakage evaluation method, resulting in 4 major categories: colorimetric, imaging, nuclear medicine, and microbiological methods. For each category, several techniques were mapped. After data collection and analysis, a narrative synthesis of the results was made.

## 3. Results

**Figure 1:**
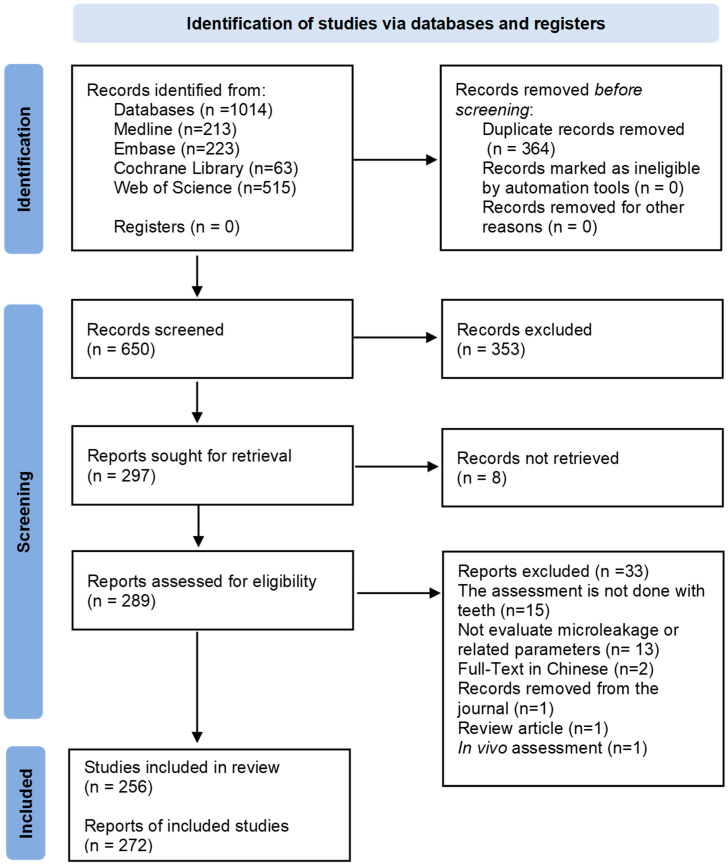
PRISMA flow diagram of the study selection.

The initial search yielded a total of 1014 potentially relevant records. After the removal of 364 duplicates, the screening based on titles and abstracts resulted in the exclusion of 353 publications. The remaining 297 records were sought for retrieval, of which 8 could not be obtained. Finally, 289 publications were submitted for full-text analysis, resulting in the exclusion of 33 records and leading to the inclusion of 256. The flow diagram depicting the review process is presented in Figure 1, and the list of excluded studies, with the respective reasons, is shown in Supplementary Material Table S3.

Geographic regions, countries, and publication language

**Figure 2:**
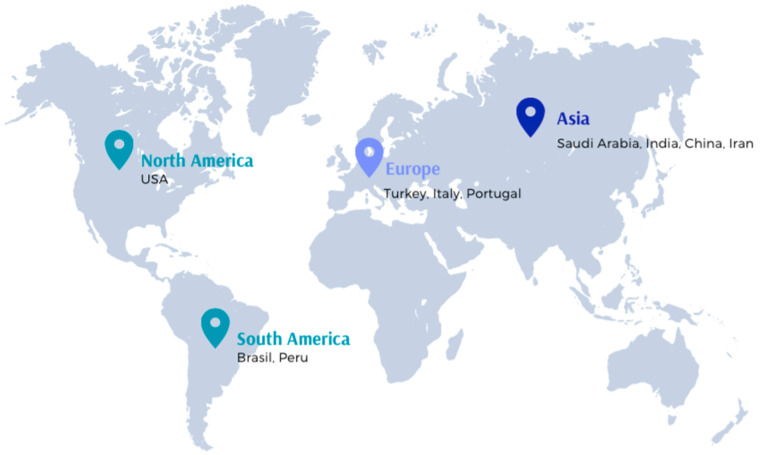
Geographic regional distribution of the included studies.

The geographic regions, countries of origin, and publication language of the included articles were automatically identified by the Rayyan software. Regarding the geographic origin, the majority of the publications were derived from Asia, followed by South America, Europe, and finally North America (Figure 2). The most productive countries, in terms of the number of published articles, were Iran (*n* = 24), Saudi Arabia (*n* = 21), and India (*n* = 20). The main languages of the included reports are English (*n* = 255) and Spanish (*n* = 1).

Publication types and journals

Considering the publication type, most of the included studies are journal articles (*n* = 241), and the remaining are book chapters (*n* = 10) and theses (*n* = 5). The journals that published the greatest number of articles included in this review were the *Dentistry Journal* (*n* = 17), *Polymers* (*n* = 13), and the *Journal of Contemporary Dental Practice* (*n* = 13). A complete list of journals and books where the included studies are published is shown in Supplementary Material Table S4.

Authorship

Regarding authorship, 248 first authors contributed to the included studies, with eight authors being the first authors of two publications. A complete list of the first authors is presented in Supplementary Table S5.

Samples and restorative procedures

The analysis of the included studies showed that the origin of the samples is mostly human teeth (*n* = 254), with only a small number being bovine teeth (*n* = 2). Molars (*n* = 104) and premolars (*n* = 82) are the most used sample types. Studies on primary dentition teeth were also included (*n* = 27). Regarding the restorative procedures, most studies use a direct technique (*n* = 218), with class V the most popular cavity shape (*n* = 83). Also, most studies submitted the samples to aging and thermocycling procedures before microleakage testing.

Microleakage evaluation methods

**Figure 3:**
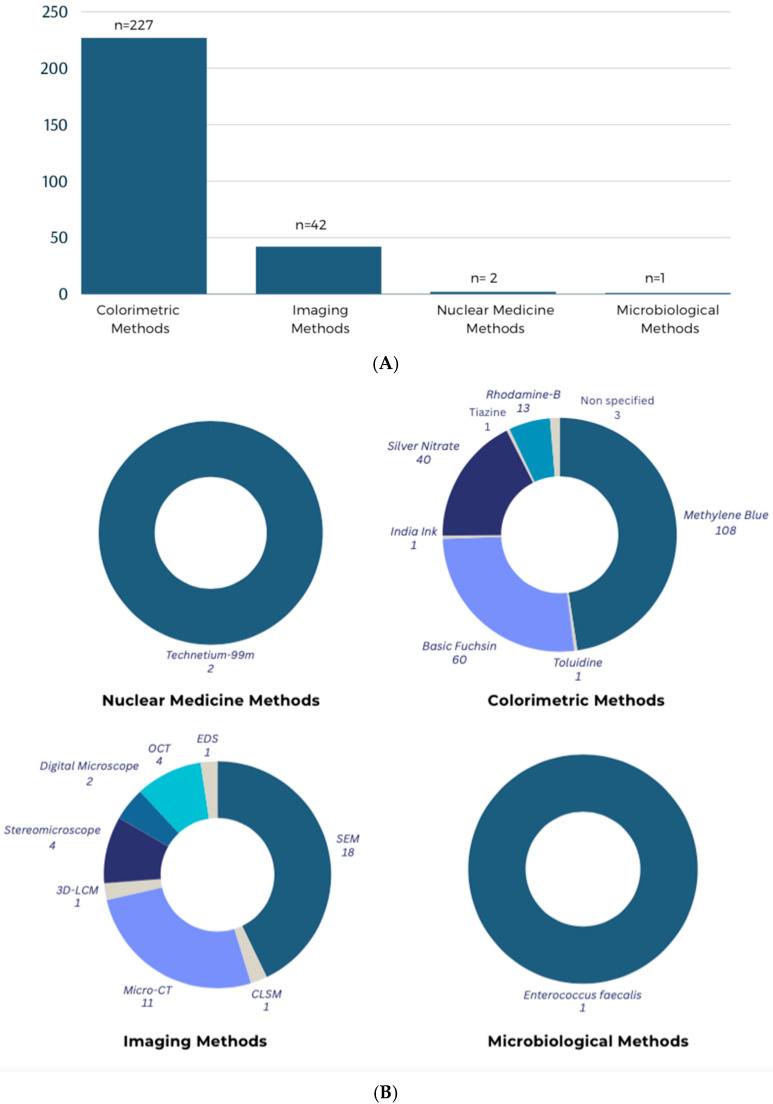
(**A**) Number of results for each category. (**B**) Evaluation techniques used and respective number of results. EDS—energy-dispersive X-ray spectrometry; OCT—optical coherence tomography; 3D-LCM—3D laser confocal microscopy; CLSM—confocal laser scanning microscopy; SEM— scanning electron microscopy; Micro-CT—micro-computed tomography.

The present review gathered 272 results describing microleakage evaluation from the 256 publications, which is because some studies report more than one method to assess microleakage. The results were grouped according to the type of assessment method: colorimetric, imaging, nuclear medicine, and microbiological methods. The number of studies in each category is presented in Figure 3A, and the evaluation techniques used are identified and enumerated in Figure 3B.

Colorimetric methods

For the colorimetric methods category, 227 results were mapped, showing different dye solutions and experimental protocols. Regarding dye solutions, the most used dye is methylene blue (*n* = 108), followed by basic fuchsin (*n* = 60), silver nitrate (*n* = 40), and rhodamine-B (*n* = 13). On the other hand, toluidine (*n* = 1), thiazine (*n* = 1), and India ink (*n* = 1) have a residual frequency of use. A summary of the information retrieved from the articles describing colorimetric methods is provided in Table 2.

The included studies unanimously describe the samples’ processing before immersion in the dye solution. All report the need to waterproof the samples with varnish, to avoid dye penetration in other areas than the restorative interface, and some studies describe sealing the apex with composite resins or similar materials to ensure good isolation [29,30].

Regarding the sample’s immersion protocol, different concentrations, immersion times, and exposition temperatures are described, as shown in Table 3. Nevertheless, the most frequently described protocols are the following: 2% methylene blue for 24 h; 0.5% basic fuchsin for 24 h; 0.1% rhodamine-B for 24 h, and 50% silver nitrate [30,31,32,33]. Regarding silver nitrate, the majority of studies report immersion in the AgNO_3_ solution to be performed in the dark, followed by washing in running water and subsequent immersion in a photo-developing solution under fluorescent light. As for the temperature, room temperature is the most used in the studies [33].

The methods used to evaluate dye penetration determine the type of assessment, which may be qualitative, semi-quantitative, or quantitative. Most of the studies make a semi-quantitative assessment (using different microleakage scoring systems), using a stereomicroscope and a microleakage scoring system previously defined [32]. When a quantitative assessment is desired, the most used method is image software systems, namely the ImageJ software. (3) Other quantitative methods to quantify dye penetration are described in Table 2 [34,35].

Finally, three articles were mapped that reported evaluating the marginal leakage using the dye penetration technique but did not specify the dye or the immersion conditions [36,37,38].

Nuclear Medicine methods

For the nuclear medicine category, two results were mapped, and both used the radioisotope Technetium-99m for the microleakage assessment, as reported in Table 3.

Again, the samples were first waterproofed with varnish and later placed in a sodium pertechnetate (99mTc-NaO_4_) solution for 3 h. After the immersion period, the varnish was completely removed, and the radioactivity of the samples was detected by a gamma camera controlled by an acquisition computer. For each sample, static images were obtained, regions of interest (ROIs) were drawn, and the total, maximum, and average counts were obtained using a specified software (XelerisTM, GE, Milwaukee, WI, USA). The total counts obtained from each image were used to quantify infiltration [20,21].

Imaging methods

The imaging methods category presents 42 results, which report eight different technologies to evaluate microleakage and the marginal misfit of the restorations. Table 4 presents a summary of the included articles’ information.

The most used method is scanning electron microscopy (SEM) (*n* = 18), with the used protocols differing essentially regarding the sample processing mode. Most studies ion sputtered the samples with gold (Au) or gold–palladium (Au-Pd) before the SEM assessment [39]. However, a few studies reported that, when SEM is used in a low vacuum mode, it allows for imaging and analysis of uncoated samples [40]. For the energy-dispersive X-ray spectroscopy (EDS), a sputter-coating with gold of the samples is also reported. For the remaining techniques, no specific sample processing is described [41].

Micro-CT (*n* = 11) is the second most used technique, since it allows for quantifying the volume and depth of microleakage [42]. Some studies reported only one scan from the restored specimen for analysis, while others made a first scan after cavity preparation, and another after the restorative procedure [43,44,45,46].

Next, stereomicroscope (*n* = 4), optical coherence tomography (*n* = 4), digital microscopy (*n* = 2), confocal laser scanning microscopy (*n* = 1), 3D laser confocal microscopy (*n* = 1), and energy-dispersive X-ray spectroscopy (*n* = 1) are also reported.

Regarding the type of evaluation, imaging methods can provide a qualitative, semi-quantitative, or quantitative assessment. For the latter, specific tools to measure microleakage or marginal discrepancy are described [41,46,47,48,49,50].

In the microbiological methods category, only one result was mapped, as shown in Table 5.

In the included study, the teeth and the system they were mounted on were previously sterilized. A simulation of Enterococcus faecalis bacterial infection was conducted for 21 days. After this period, the samples were sectioned and stained using a Live/Dead BacLight Bacterial Viability Kit L-7012. The fluorescence from the stained bacteria was observed under a confocal laser scanning microscope. The extent of fluorescent staining within the evaluated areas was calculated using appropriate software, and the distance between the bacterial load in the crown area and the pulp chamber was measured [51].

A summary of the information retrieved from the 272 results is provided in Table S6.

## 4. Discussion

The present scoping review was performed to map and summarize the existing current evidence about the microleakage assessment techniques and their methodologies. The central question of this review was what microleakage assessment methods are currently used in the evaluation of coronary restorative materials? According to the present results, the methods described are dye penetration, nuclear medicine using radioactive isotopes, bacteria infiltration, and imaging methods, presenting variations in their methodologies. The literature reports other in vitro methods for microleakage assessment that are not contemplated in this review, as they do not satisfy the inclusion criteria. Glucose penetration, protein infiltration, electrochemical methods, air pressure, and neutron activation analysis are most employed in microleakage evaluation in endodontic studies and have also been mostly replaced by more contemporary methods [4,30,42,52,53].

According to the included reports, the most employed technique is dye penetration using colored agents, which is consistent with the actual evidence [30,54]. Several reasons are presented by the studies to support the choice for such a technique: easily performed, low cost, simple to replicate, and no specific equipment, radiation, or reactive chemicals are employed [30,54,55]. However, in addition to the inherent limitations of this technique, the non-standardization of methodologies compromises the comparison of results [56]. The experimental protocols differ in the type of dye used and the immersion conditions (concentration, time, and temperature), which can lead to differences in the degree of dye penetration [56]. Sample processing is also debatable, with the majority of studies describing sectioning the specimens in the center of restoration, which results in only two interfaces for evaluation, while a few others promote several sections, allowing for choosing the most infiltrated among the various sections. However, it is described that the simple sectioning of the specimens in their center shows lower infiltration values in comparison with multiple sections [56]. Another technique introduced by Gale and Darvell involves grinding the specimen into sequential slices and then reconstructing the images using software, which can surpass the limitations of just evaluating a few slices [17]. However, this method is only employed in a limited number of studies. Regarding the type of assessment, the semi-quantitative method is the most used, despite quantitative methods being available that could provide more reliable information. This way, quantitative methods should be employed more often, such as quantifying the amount of dye penetration using spectrophotometry [34,35]. Other quantitative methods were also mapped, which are not examiner-dependent and represent valuable options. Finally, the specimen analysis, to confirm that the penetration dye occurred through the tooth/restorative material interface and not through another region, is fundamental to validate the results [56].

Methylene blue was the most used dye in the included studies. This preference can be justified by the fact that it is easily observable, diffuses through contact, is not absorbable by dentinal matrix hydroxyapatite crystals, and presents good penetrability and void penetration [30,34,54,55]. However, there are significant differences in dye concentration, soaking time, and temperature in colorimetric methods (such as methylene blue concentration ranging from 0.1% to 10%), which limit the comparability of the results. Regarding the use of silver nitrate solutions, the strong optical contrast of silver particles, as well as the use of developer solution (which causes the aggregation of silver, increasing the amount of microleakage detected), makes it especially interesting not only for direct evaluation but also for combination with other techniques, such as micro-CT [52,57]. Also, an evaluation method using energy-dispersive analytical X-ray spectrometry (EDAX) allows for quantification of the amount of silver grain deposition at a nanoscale level and provides precise results [33].

Imaging methods were the second category with the most results. In this category, eight technologies were mapped, which allows for a two-dimensional (2D) or three-dimensional (3D) analysis of the restorative interface. Microleakage is a three-dimensional (3D) phenomenon, so the 2D evaluation, frequently based on a single longitudinal slice, can underestimate or overestimate the actual microleakage penetration along a margin length and should be avoided [46]. Scanning electron microscopy (SEM) allows for observing with accuracy the marginal adaptation of a restorative material with the cavity margin, but does not quantify the diffusion or penetration of microleakage, which is a limitation [52]. For SEM assessment, the literature reports two methodologies, namely the direct technique and the replica technique [52]. The direct technique involves using the specimen itself for microscopic evaluation, running the risk of introducing artifacts during the sample processing for imaging. The replica technique allows for overcoming this problem, since impressions are taken of the samples and subsequently filled for the analysis, resulting in a high-resolution replica that reflects the microstructural details of the specimens [52]. Regarding the studies included in this review, they used the direct technique. Thus, most studies describe ion sputtering of the samples with gold or Au-Pd using ion-coating equipment before the SEM assessment [39,50]. However, a few studies reported that, when SEM is used in a low vacuum mode, it allows for imaging and analysis of uncoated samples, which is less technical, time-consuming, and cheaper [40].

Micro-computed tomography (micro-CT) creates a 3D visualization of dental structures from the reconstruction of 2D images, allowing void visualization and quantitative measurements, such as the width and the volume of the gap [42]. For this evaluation, the methodologies differ essentially in number and the moment at which the scans were taken. In some studies, one scan is taken from the restored specimen and analyzed, while in others, they make a first scan after cavity preparation and another after the restorative procedure. Superimposing the two images in specific software allows for the reconstruction and subtraction of the images and detailed measurements [43,44,45,46]. Also, Putignano et al. used EDS for validating the gaps and misfits found in the micro-CT reconstruction model, since it allows for the identification of the elemental composition of materials, determining if the voids consist of gaps or an adhesive layer [41].

Optical coherence tomography (OCT) produces 3D images and evaluates microleakage localization, continuity, and gap width between the composite resin restoration and the substrate [39,42,47,58,59]. The technologies used were spectral-domain optical coherence tomography (SD-OCT) and cross-polarization optical coherence tomography (CP-OCT) [60,61]. In both technologies, the restoration should be perpendicular to the light beam in such a way that the infrared beam traverses over the tooth surface. The studies included do not present differentiating parameters, except with regard to the settings, which limit replicability. Nevertheless, OCT has a measurement depth limit of around 2–3 mm in many tissues, which might affect the results and may not be appropriate in microleakage studies [39,47,58,59].

Regarding stereomicroscope and digital microscope technologies, they allow for observing in 2D the marginal gap between the tooth structure and the restorative material. After this, the images can be measured with image-specific software programs, allowing quantification [62,63,64].

The use of radioactive isotopes allows for the detection and quantification of infiltration, even at very small concentrations [20,21]. In the included studies, Technetium-99m was used for the microleakage assessment. In this evaluation, the radioactivity detected by a gamma camera corresponds to the microleakage in areas of the adhesive interface that allow technetium penetration [20,21]. The choice of the Technetium-99m radionuclide is due to its selectivity, traveling through the tooth by capillarity and depositing in the gap areas.

Also, its smaller molecular size is comparable to that of the microorganisms present in saliva (simulating the clinical conditions of bacterial infection) and allows for the evaluation of the same sample at different time points, since the samples are not destroyed and only energy is dissipated, while the molecule remains stable [65]. Other studies also report the use of Ca45. However, some authors reported that its affinity for restorative materials and tooth structure may lead to measurement errors [22,52,56,66]. Despite accurate quantitative measures and the high sensitivity of the technique, the necessity of specific equipment and radiopharmaceutical manipulation, and availability limits its use [65].

Microbiological models to assess microleakage may be the most clinically relevant ones [54]. This method requires a controlled sterile environment to avoid contamination with other bacteria and validate the results for the chosen strain [51,52]. In the study mapped, a simulation of Enterococcus faecalis bacterial infection was conducted for 21 days.

Enterococcus faecalis was the chosen strain since this species exists in the normal oral flora in humans, is frequently found in mixed infections with other aerobes and facultative anaerobes, does not form endospores, and plays an essential role in bacterial biofilm formation. Therefore, it is considered an appropriate model for testing novel treatments. The subsequent evaluation with CLSM provided direct and quantifiable information about the presence and distribution of live and dead E. faecalis bactéria inside the dentinal tubules, which allowed for tracking and quantifying the extent of microleakage [51].

Some studies used more than one technique for microleakage assessment. The association of techniques allows for achieving more reliable and debatable results in microleakage studies [13,39,41,45,51,55,58,67].

As previously referred, the included studies present different experimental protocols for the same technique. The protocols differ essentially regarding the sample-processing mode, the study variables, and the type of assessment that is conducted. Thus, differences in the results have been attributed to differences in the methodology used and the sensitivity of the tests [52,56]. In this sense, the authors refer to the standardization of in vitro conditions as a fundamental step to control possible bias factors, optimize the statistical analysis, and allow for reproducibility [20,52,56]. Also, it is fundamental that the studies report all the information regarding the methodology and materials used, which was not the case in some of the included reports, which compromises the reproducibility. Lastly, the restorative procedures should be performed by the same operator to reduce the associated human error [20].

In vitro studies allow for the performance of single-variable experiments under controlled conditions, providing important conclusions. However, their reductionist approach requires careful consideration of their limitations, as in vitro systems cannot replicate a dynamic environment, such as the stomatognathic system [68]. Nevertheless, most of the studies included in this review subjected the samples to thermocycling before microleakage testing to simulate the oral environment. Still, the studies highlight that long-term clinical studies are necessary to consolidate the results.

As for the limitations of the scoping review, we can mention that it only includes articles from the last 5 years. Although the goal of the review was to map the currently used methods, the inclusion of more articles could increase the information obtained and allow for some methods to have more included results. Also, only the first authors were counted, which may underrepresent the most prolific authors in this field.

Although this scoping review does not provide a critical synthesis comparing the accuracy, repeatability, cost, and clinical relevance of each method, its purpose was to map the current landscape of microleakage assessment techniques rather than to evaluate their relative performance. Nonetheless, the data collected highlight important trends that merit further exploration in future systematic reviews. For instance, colorimetric methods remain dominant likely due to their simplicity, low cost, and accessibility, particularly in resource-limited settings. However, advancements in imaging technologies and nuclear medicine are progressively enhancing the precision and depth of microleakage evaluation, suggesting a gradual shift toward more sophisticated, albeit more costly, diagnostic tools. These evolving trends underscore the need for standardized protocols and comparative studies to guide researchers and clinicians in selecting the most appropriate and clinically relevant methods [65].

## 5. Conclusions

Currently, several microleakage assessment techniques are being used. The dye penetration method with colorimetric agents is the most widely used, followed by imaging techniques, nuclear medicine, and microbiological models. Regarding the dyes, methylene blue is the most used one. However, there is a lack of consistency in the methodologies used, which contributes to the variability of results and the difficulty in comparing the results.

Regarding the in vitro investigation, the obtained results show that the use of dyes and nuclear medicine methods is sensitive and can be used to evaluate microleakage.

Nonetheless, differences persist in the types of dyes used and in application protocols. Moreover, emerging three-dimensional (3D) assessment techniques show promise in improving the accuracy and reliability of microleakage evaluation by providing more comprehensive and spatially detailed analysis.

Given the predominance of studies from specific geographic regions—particularly Iran, Saudi Arabia, and India—questions remain about the global applicability of the findings. This reinforces the relevance of our scoping review, which aims to map the techniques currently in use rather than compare their effectiveness.

In light of the observed methodological heterogeneity, the establishment of standardized guidelines for microleakage testing is strongly recommended. Such guidelines would enhance the comparability, reproducibility, and overall quality of future studies, ultimately contributing to more robust evidence for clinical application.

## Figures and Tables

**Table 1:** Eligibility Criteria.

Inclusion Criteria	Exclusion Criteria
Coronary structure restoration	Microleakage evaluation in fields other than restorative dentistry
Temporary or definite restorative material	Other study types
Teeth	
In vitro/Ex vivo studies	

**Table 2:** Summary of colorimetric methods information.

Colorimetric Methods (*n* = 227)		
**Dye**	**Protocol**	**Type of Assessment**
**Methylene Blue (*n* = 108)**	**Concentrations**: 0.1%, 5%, 1%, 2%, 5%, and 10% **Exposure Time**: 2 h, 4 h, 12 h, 24 h, 48 h, and 72 h **T(°C)**: Room temperature or 37 °C	**Qualitative** **Semi-Quantitative**: Microscopy and microleakage scoring systems. **Quantitative**: • ELISA • Spectrophotometer • Microscopy and image analysis software (ImageJ, version 1.54p, Omnimet)
**Basic Fuchsin** **(*n* = 60)**	**Concentrations**: 0.5%, 2% **Exposure Time**: 8 h, 24 h, 48 h, and 7 days. **T(°C)**: Room temperature or 37 °C	**Qualitative** **Semi-Quantitative**: Microscopy and microleakage scoring systems. **Quantitative**: Microscopy and image analysis software (ImageJ, QuickPhoto Micro 2.2, DinLightht Pro, Image Pro Plus)
**Silver Nitrate** **(*n* = 40)**	**Formulations:** 50% wt% or 50% ammoniacal AgNO_3_ **T(°C)**: room temperature or 37 °C **Exposure Conditions:** Only in AgNO_3_ solution for 24 h. AgNO_3_ solution in the dark + Photodeveloping solution under fluorescent light: (24 h + 8 h), (24 h + 12 h), (24 h + 6 h), and (12 h + 8 h).	**Qualitative** **Semi-Quantitative:** Microscopy and microleakage scoring systems. **Quantitative**: • Micro-CT. • Microscopy and image analysis software (ImageJ, Optimas 6.51). • SEM and energy-dispersive analytical X-ray spectrometry assessment.
**Rhodamine-B** **(*n* = 13)**	**Concentrations**: 0.02%, 0.1%, 0.2%, 0.5%, and 1%. **Exposure Time**: 10 h, 24 h, and 48 h. **T(°C)**: Room temperature or 37 °C	**Semi-quantitative**: Microscopy and microleakage scoring systems. **Quantitative**: Confocal laser scanning microscope and ImageJ software.
**Toluidine** **(*n* = 1)**	**Concentration:** 1% **Exposure Time:** 24 h **T(°C):** room temperature	**Semi-Quantitative:** Microscopy and microleakage scoring systems
**India Ink** **(*n* = 1)**	**Concentrations:** non-specified **Exposure Time:** 24 h **T(°C):** 37 °C	**Semi-quantitative:** Microscopy and microleakage scoring systems.
**Thiazine** **(*n* = 1)**	**Concentrations:** 2% **Exposure Time:** 24 h **T(°C):** 37°	**Quantitative:** Microscopy methods and image analysis software (Optika Vision lite 2.1 software)

T(°C)—temperature; AgNO_3_—silver nitrate; Micro-CT—micro-computed tomography; SEM—scanning electron microscopy.

**Table 3:** Summary of nuclear medicine methods information.

Nuclear Medicine Method (*n* = 2)		
Radioisotope	Protocol	Type of Assessment
**Technetium-99m** **(*n* = 2)**	Immersion of the samples in a sodium pertechnetate (99mTc-NaO_4_) solution for 3 h. The radioactivity of the samples was detected by a gamma camera.	**Quantitative** The radioactivity emitted by the samples was detected by a gamma camera. A static image was obtained for each specimen. The total counts obtained from each image were used to quantify infiltration.

**Table 4:** Summary of imaging methods information.

Imaging Methods (*n* = 42)		
**Imaging Method**	**Variable of the Protocol**	**Type of Assessment**
**SEM** **(*n* = 18)**	**Sample Processing:** • Ion sputtered the samples with gold or Au-Pd using ion coating equipment before the SEM assessment • SEM low vacuum mode allows imaging and analysis of uncoated samples	**Qualitative** **Semi-quantitative**: microleakage scoring systems **Quantitative:** ImageJ analysis software, VGSTUDIO MAX, NRecon
**Micro-CT** **(*n* = 11)**	**Number and moment of scans:** • Only one scan from the restored specimen • Scan after cavity preparation and another scan after restorative treatment or with other variables (before/after light curing, etc)	**Quantitative** • Specific tools from the Micro-CT allow for measuring the volume of the gaps. • Image analysis software (ImageJ, VGSTUDIO MAX, NRecon)
**Stereomicroscope** **(*n* = 4)**	Direct observation	**Qualitative** **Quantitative**: Image analysis software (NIS-Element’s viewer, AxioVision)
**Optical Coherence Tomography** **(*n* = 4)**	**Technologies:** • Spectral-domain optical coherence tomography (SD-OCT) • Cross-polarization optical coherence tomography (CP-OCT) Differences in settings	**Quantitative**: ImageJ analysis software
**Digital Microscope** **(*n* = 2)**	Direct observation	**Qualitative** **Quantitative:** ImageJ analysis software
**Confocal Laser Scanning Microscopy (CLSM)** **(*n* = 1)**	Direct observation and measurement	**Quantitative**: Specific tools from the CLSM allow the measurement of the perimeter of the tooth–restoration interface and the sum of the gaps.
**3D-Laser Confocal Microscopy** **(*n* = 1)**	Direct observation and measurement	**Quantitative**: Specific tools from the 3D-LCM allow the identification of areas with gaps in the tooth composite interface (height filter) and measure the length of the gaps (linear marker)
**Energy Dispersive** **X-ray Spectroscopy** **(*n* = 1)**	**Sample processing:** Ion sputtered the samples with gold ion coating equipment before the EDS assessment	**Qualitative**: X-ray technique used to identify the elemental composition of materials, allowing confirmation if the voids consist of gaps or adhesive layer.

3D-LCM—3D laser confocal microscopy; CLSM—confocal laser scanning microscopy; SEM—scanning electron microscopy; Micro-CT—micro-computed tomography; EDS—energy-dispersive X-ray spectrometry.

**Table 5:** Summary of microbiological methods information.

Microbiological Method (*n* = 1)		
Strain	Protocol	Type of Assessment
**Enterococcus faecalis** **(*n* = 1)**	*Enterococcus faecalis* bacterial infection for 21 days. After this period, the samples were sectioned and stained using a Live/Dead Backlight Bacterial Viability Kit L-7012. The fluorescence from the stained bacteria was observed and measured with CLSM.	**Quantitative** Using a confocal laser scanning microscope, it is possible to track and quantify the routes and extent of bacterial colonization and therefore measure the extent of microleakage.

CLSM—confocal laser scanning microscopy.

## Data Availability

No new data were created or analyzed in this study. Data sharing is not applicable to this article.

## Supplementary Materials

The following supporting information can be downloaded at https://www.mdpi.com/article/10.3390/jfb16060210/s1, Table S1: PRISMA checklist; Table S2: Databases search strategy and filter used; Table S3: List of excluded articles by full-text and respective reason; Table S4: List of the journals and respective number of published studies included in this review; Table S5: List of the first authors that contribute to the included studies; Table S6: Summary of the information retrieved from the 272 results.
