# Supplementary material for: In Vitro Techniques for Microleakage Evaluation of Coronary Restorative Materials: A Scoping and Mapping Review
Source: J Funct Biomater. 2025 Jun 4;16(6):210. doi: 10.3390/jfb16060210 (PMC12194229; doi:10.3390/jfb16060210)
Supplement: Supplementary file 1 [file jfb-16-00210-s001.zip › jfb-3617543-supplementary.pdf]

**Supplemmentary Materials**

**Table S1:** PRISMA checklist.

| Section And Topic | Item # | Checklist Item | Location where item is reported |
|---|---|---|---|
| **TITLE** | | | |
| Title | 1 | Identify the report as a systematic review. | Cover |
| **ABSTRACT** | | | |
| Abstract | 2 | See the PRISMA 2020 for Abstracts checklist. | 7 |
| **INTRODUCTION** | | | |
| Rationale | 3 | Describe the rationale for the review in the context of existing knowledge. | 11 |
| Objectives | 4 | Provide an explicit statement of the objective(s) or question(s) the review addresses. | 13 |
| **METHODS** | | | |
| Eligibility criteria | 5 | Specify the inclusion and exclusion criteria for the review and how studies were grouped for the syntheses. | 15 |
| Information sources | 6 | Specify all databases, registers, websites, organisations, reference lists and other sources searched or consulted to identify studies. Specify the date when each source was last searched or consulted. | 14 |
| Search strategy | 7 | Present the full search strategies for all databases, registers and websites, including any filters and limits used. | 53 |
| Selection process | 8 | Specify the methods used to decide whether a study met the inclusion criteria of the review, including how many reviewers screened each record and each report retrieved, whether they worked independently, and if applicable, details of automation tools used in the process. | 15 |
| Data collection process | 9 | Specify the methods used to collect data from reports, including how many reviewers collected data from each report, whether they worked independently, any processes for obtaining or confirming data from study investigators, and if applicable, details of automation tools used in the process. | 15 |
| Data items | 10a | List and define all outcomes for which data were sought. Specify whether all results that were compatible with each outcome domain in each study were sought (e.g. for all measures, time points, analyses), and if not, the methods used to decide which results to collect. | 15 |
| | 10b | List and define all other variables for which data were sought (e.g. participant and intervention characteristics, funding sources). Describe any assumptions made about any missing or unclear information. | 14 |
| Study risk of bias assessment | 11 | Specify the methods used to assess risk of bias in the included studies, including details of the tool(s) used, how many reviewers assessed each study and whether they worked independently, and if applicable, details of automation tools used in the process. | |
| Effect measures | 12 | Specify for each outcome the effect measure(s) (e.g. risk ratio, mean difference) used in the synthesis or presentation of results. | 14 |
| Synthesis methods | 13a | Describe the processes used to decide which studies were eligible for each synthesis (e.g. tabulating the study intervention characteristics and comparing against the planned groups for each synthesis (item #5)). | 15 |
| | 13b | Describe any methods required to prepare the data for presentation or synthesis, such as handling of missing summary statistics, or data | 15 |

| | | | |
|---|---|---|---|
| | | conversions. | |
| | 13c | Describe any methods used to tabulate or visually display results of individual studies and syntheses. | 15 |
| | 13d | Describe any methods used to synthesize results and provide a rationale for the choice(s). If meta-analysis was performed, describe the model(s), method(s) to identify the presence and extent of statistical heterogeneity, and software package(s) used. | 15 |
| | 13e | Describe any methods used to explore possible causes of heterogeneity among study results (e.g. subgroup analysis, meta-regression). | 15 |
| | 13f | Describe any sensitivity analyses conducted to assess robustness of the synthesized results. | 15 |
| Reporting bias assessment | 14 | Describe any methods used to assess risk of bias due to missing results in a synthesis (arising from reporting biases). | |
| Certainty assessment | 15 | Describe any methods used to assess certainty (or confidence) in the body of evidence for an outcome. | |
| **RESULTS** | | | |
| Study selection | 16a | Describe the results of the search and selection process, from the number of records identified in the search to the number of studies included in the review, ideally using a flow diagram. | 22 |
| | 16b | Cite studies that might appear to meet the inclusion criteria, but which were excluded, and explain why they were excluded. | 22 |
| Study characteristics | 17 | Cite each included study and present its characteristics. | |
| Risk of bias in studies | 18 | Present assessments of risk of bias for each included study. | |
| Results of individual studies | 19 | For all outcomes, present, for each study: (a) summary statistics for each group (where appropriate) and (b) an effect estimate and its precision (e.g. confidence/credible interval), ideally using structured tables or plots. | 23-32 |
| Results of syntheses | 20a | For each synthesis, briefly summarise the characteristics and risk of bias among contributing studies. | |
| | 20b | Present results of all statistical syntheses conducted. If meta-analysis was done, present for each the summary estimate and its precision (e.g. confidence/credible interval) and measures of statistical heterogeneity. If comparing groups, describe the direction of the effect. | 23-32 |
| | 20c | Present results of all investigations of possible causes of heterogeneity among study results. | 23-32 |
| | 20d | Present results of all sensitivity analyses conducted to assess the robustness of the synthesized results. | 23-32 |
| Reporting biases | 21 | Present assessments of risk of bias due to missing results (arising from reporting biases) for each synthesis assessed. | |
| Certainty of evidence | 22 | Present assessments of certainty (or confidence) in the body of evidence for each outcome assessed. | |
| **DISCUSSION** | | | |

| | | | |
|---|---|---|---|
| Discussion | 23a | Provide a general interpretation of the results in the context of other evidence. | 36-41 |
| | 23b | Discuss any limitations of the evidence included in the review. | 36-41 |
| | 23c | Discuss any limitations of the review processes used. | 36-41 |
| | 23d | Discuss implications of the results for practice, policy, and future research. | 36-41 |
| **OTHER INFORMATION** | | | |
| Registration and protocol | 24a | Provide registration information for the review, including register name and registration number, or state that the review was not registered. | 14 |
| | 24b | Indicate where the review protocol can be accessed, or state that a protocol was not prepared. | 14 |
| | 24c | Describe and explain any amendments to information provided at registration or in the protocol. | |
| Support | 25 | Describe sources of financial or non-financial support for the review, and the role of the funders or sponsors in the review. | |
| Competing interests | 26 | Declare any competing interests of review authors. | |
| Availability of data, code and other materials | 27 | Report which of the following are publicly available and where they can be found: template data collection forms; data extracted from included studies; data used for all analyses; analytic code; any other materials used in the review. | 14-15 |

**Table S2:** Databases search strategy and filter used.

| | |
|---|---|
| **Medline (through PubMed)** | ("Dental Leakage"[Mesh] OR leakage* OR microleakage OR shrinkage) AND (evaluat* OR assess*) AND ("Tooth"[Mesh] OR tooth OR teeth) NOT ("Endodontics"[Mesh] OR endodontic* OR “root canal” OR “canal, dental root” OR “canal, tooth root” OR “canalis radicis dentis” OR “dental canal” OR "Orthodontic Appliances"[Mesh] OR "Orthodontics, Corrective"[Mesh] OR orthosystem OR “dental appliance*” OR "Orthodontic Brackets"[Mesh] OR bracket* OR brace* OR "Dental Implants"[Mesh] OR implant*)<br><br>Language Filter: English, French, Spanish or Portuguese<br>Temporal Filter: 2020-2024<br>Number of retrieved articles: 213 |
| **Embase** | ('dental leakage' OR leakage* OR 'microleakage'/exp OR 'microleakage' OR 'shrinkage'/exp OR 'shrinkage') AND ('evaluation study'/exp OR 'evaluat*' OR 'assessment'/exp OR assess*) AND ('tooth'/exp OR 'dentes' OR 'teeth' OR 'tooth') NOT ('endodontics'/exp OR endodontic* OR 'tooth root canal'/exp OR 'canal, dental root' OR 'canal, tooth root' OR 'canalis radicis dentis' OR 'dental canal' OR 'root canal' OR 'orthodontic device'/exp OR 'orthosystem' OR 'dental appliance' OR 'dental appliances' OR 'tooth brace' OR bracket* OR 'tooth implant'/exp OR 'implant*') AND ([english]/lim OR [french]/lim OR [portuguese]/lim OR [spanish]/lim) AND ([article]/lim OR [article in press]/lim OR [data papers]/lim OR [editorial]/lim OR [erratum]/lim OR [review]/lim OR [short survey]/lim)<br><br>Temporal Filter: 2020-2024<br>Number of retrieved articles: 223 |
| **Cochrane Library** | ([Dental Leakage] OR leakage* OR microleakage* OR shrinkage) AND ([Evaluation Study] OR evaluat* OR assess*) AND ([Tooth] OR tooth OR teeth) NOT ([Endodontics] OR endodontic* OR "root canal" OR "canal, dental root" OR "canal, tooth root" OR "canalis radicis dentis" OR "dental canal" [Orthodontic Appliances] OR [Orthodontics, Corrective] OR orthosystem OR (dental NEXT appliance*) OR [Orthodontic Brackets] OR bracket* OR (tooth NEXT brace*) OR [Dental Implants] OR implant*)<br><br>Temporal Filter: 2020-2024<br>Number of retrieved articles: 63 |
| **Web of Science (all databases)** | (leakage* OR microleakage OR shrinkage) AND (evaluat* OR assess*) AND (tooth OR teeth) NOT (endodontic* OR “root canal” OR “tooth root canal” OR “canal, dental root” OR “canal, tooth root” OR “canalis radicis dentis” OR “dental canal” OR orthosystem OR “dental appliance*” OR bracket* OR brace* OR implant*)<br><br>Language Filter: English, Spanish, Portuguese or Unspecified<br>Temporal Filter: 2020-2024 |

NOT Document Types: Review Article or Meeting or Awarded Grant or Abstract or Book
Number of retrieved articles: 515

Table S3: List of excluded articles by full-text and respective reason.

| Publication Title | Author and Year | Reasons for exclusion |
|---|---|---|
| **1.** Prevention of Bacterial Infiltration in Class 1 Temporary Restorations Using Zinc Oxide/Calcium Sulphate Filling Materials: An *In Vitro* Study | Buonavoglia *et al.* (2023) | The assessment is not done in teeth |
| **2.** Marginal Adaptation of *in vitro* Class II Restorations Made Out of Bulk or Conventional Composite Using Single- or Multi-Layered Techniques | Dietschi *et al.* (2023) | |
| **3.** Wear and marginal quality of aesthetic crowns for primary molars | Moehn *et al.* (2022) | |
| **4.** Microtensile Bond Strength, Bonding Interface Morphology, Adhesive Resin Infiltration, and Marginal Adaptation of Bulk-fill Composites Placed Using Different Adhesives | Sebold *et al.* (2021) | |
| **5.** *In vitro* Evaluation of the Marginal and Internal Accuracy of Different Types of Dental Ceramic Restorations Fabricated Based on Digital and Conventional Impressions | Ozsurmeli *et al.* (2021) | |
| **6.** *In vitro* evaluation of marginal adaptation in medium- and large size direct class II restorations using a bulk-fill or layering technique | Dietschi *et al.* (2021) | |
| **7.** Evaluation of low-viscosity bulk-fill composites regarding marginal and internal adaptation | Park *et al.* (2021) | |
| **8.** Polymerization Stress and Gap Formation of Self-adhesive, Bulk-fill and Flowable Composite Resins | Nakano *et al.* (2020) | |
| **9.** Ceramic laminate veneers: effect of preparation design and ceramic thickness on fracture resistance and marginal quality *in Vitro* | Blunck *et al.* (2020) | |
| **10.** *In Vitro* Comparison of the physical and mechanical properties of an ormocer with an ormocer-based composite and a nanocomposite restorative material. | *Jansen van Rensburg et al.* (2023) | |

| | | | |
|---|---|---|---|
| **11.** | An *In-Vitro* Evaluation of Microleakage in Resin-Based Restorative Materials at Different Time Intervals | Bilgrami *et al.* (2022) | |
| **12.** | Comparative Evaluation of Compressive Strength, Microleakage, Fluoride Release and Recharge Ability of Various Glass Ionomer Based Restorative Materials: An In Vitro Study | Henna Basheer *et al.* (2020) | |
| **13.** | Marginal and internal fit of provisional crowns fabricated using 3D printing technology. | Chaturvedi *et al.* (2020) | |
| **14.** | A comparison of the marginal gaps of lithium disilicate crowns fabricated by two different intraoral scanners | Kwong *et al.* (2020) | |
| **15.** | Effect of Chairside CAD/CAM Restoration Type on Marginal Fit Accuracy: A Comparison of Crown, Inlay and Onlay Restorations | Merril *et al.* (2021) | |
| **16.** | Internal Adaptation of Cusp-weakened Class I Preparations Restored with Bulk-fill, Bi-layered, and Incremental Restorative Techniques: A Micro-CT Analysis | Floriani *et al.* (2022) | Did not evaluate microleakage or related parameters |
| **17.** | Novel antibacterial low-shrinkage-stress resin-based cement | *Al Sahafi et al.* (2022) | |
| **18.** | Novel Bio-Interactive Fixed Dental Restoration Cement with Potent Antibacterial and Remineralization Properties | *Al Sahafi et al.* (2022) | |
| **19.** | Effect of titanium dioxide nanotubes on the mechanical and antibacterial properties of the low-viscosity bulk-fill composite | Ozlen *et al.* (2022) | |
| **20.** | Inhibition of the Biofilm Formation of Anaerobic Bacteria Involved in Secondary Caries by Dental Adhesive | Thaweboon *et al.* (2023) | |
| **21.** | Evaluation of Antibacterial Activity of a Bioactive Restorative Material Versus a Glass-Ionomer Cement on Streptococcus Mutans: *In-Vitro* Study | Conti *et al.* (2023) | |
| **22.** | Microbiological and SEM assessment of atraumatic restorative treatment in adult dentition | Atay *et al.* (2021) | |
| **23.** | Noninvasive Adaptation Appraisal of Antimicrobial Nano-Filled Composite | Naguib *et al.* (2023) | |
| **24.** | Internal Adaptation of Composite Fillings Made Using Universal Adhesives-A Micro-Computed Tomography Analysis | Kaczor-Wiankowska *et al.* (2024) | |
| **25.** | The assessment of internal adaptation and fracture resistance of glass ionomer and resin-based restorative | *Demirel et al.* (2023) | |

| | | | |
|---|---|---|---|
| | materials applied after different caries removal techniques in primary teeth: an *in-vitro* study | | |
| **26.** | Comparison of Different Dentin Deproteinizing Agents on Bond Strength and Microleakage of Universal Adhesive to Dentin | Bedir *et al.* (2023) | |
| **27.** | Crack propensity of different direct restorative procedures in deep MOD cavities | *Néma et al.* (2023) | |
| **28.** | Bacteria in the cavity-restoration interface after varying periods of clinical service - SEM description of distribution and 16S rRNA gene sequence identification of isolates | Arora *et. al.* (2022) | |
| **29.** | Effects of thermal cycling on bonding properties of novel low-shrinkage resin adhesive. | Wang *et al.* (2023) | Full-Text in chinese |
| **30.** | Comparison of Microleakage and Compressive Strength of Different Base Materials | Young *et al.* (2021) | |
| **31.** | Impact of riboflavin and curcumin mediated photodynamic therapy and Er,Cr:YSGG on microleakage of class V resin glass ionomer restorations in permanent molar teeth. | Aljamhan *et al.* (2021) | Removed from the jornal |
| **32.** | Effect of Dentin Conditioning on Bonding and Sealing Ability of Various Resin Cements to a Ceramic Crown | Al Ghamdi *et al* (2021) | Review article |
| **33.** | Analysis of Weighted Fraction of Length for Interfacial Gap in Cervical Composite Restorations as a Function of the Number of B-Scans of OCT Volume Scans | *Schneider et al.* (2021) | *In vivo assessment* |

**Table S4.** List of the journals and respective number of published studies included in this review.

| *Journal* | *Number of articles included in this review* |
|---|---|
| *Dental Materials* | 17 |
| *Polymers* | 13 |
| *The Journal of Contemporary Dental Practice* | 13 |
| *International Journal of Clinical Pediatric Dentistry* | 11 |
| *BMC Oral Health* | 9 |
| *Journal of Clinical and Diagnostic Research* | 7 |
| *Journal of Pharmacy and Bioallied Sciences* | 7 |
| *Materials* | 7 |
| *Photodiagnosis and Photodynamic Therapy* | 7 |
| *Journal of Prosthetic Dentistry* | 5 |
| *Applied Sciences-Basel* | 4 |
| *Clinical and Experimental Dental Research* | 4 |
| *Dental Materials* | 4 |
| *European Archives of Pediatric Dentistry* | 4 |
| *Indian Journal of Dental Research* | 4 |
| *Journal of the Mechanical Behavior of Biomedical Materials* | 4 |
| *Journal of Clinical and Experimental Dentistry* | 4 |
| *Journal of Clinical Pediatric Dentistry (JOCPD)* | 4 |
| *Operative Dentistry* | 4 |
| *Saudi Dental Journal* | 3 |
| *Journal of Lasers in Medical Sciences* | 3 |
| *Journal of International Oral Health* | 3 |
| *European Oral Research* | 3 |
| *Cureus Journal of Medical Science* | 3 |
| *Biomaterial Investigations in Dentistry* | 3 |
| *BioMed Research International* | 3 |
| *Journal of Research in Medical and Dental Science* | 3 |
| *American Journal of Dentistry* | 3 |

| | |
|---|---|
| *Contemporary Clinical Dentistry* | 2 |
| *Clinical, Cosmetic and Investigational Dentistry* | 2 |
| *European Journal of Oral Sciences* | 2 |
| *Heliyon* | 2 |
| *International Journal of Dentistry* | 2 |
| *Indian Journal of Forensic Medicine & Toxicology* | 2 |
| *Journal of the Indian Society of Pedodontics and Preventive Dentistry* | 2 |
| *Journal of International Society of Preventive and Community Dentistry* | 2 |
| *Journal of Dental Research* | 2 |
| *Journal of Biomedical Research* | 2 |
| *Journal of Applied Oral Science* | 2 |
| *Pesquisa Brasileira em Odontopediatria e Clínica Integrada* | 2 |
| *Acta Odontologica Latinoamericana* | 2 |
| *Annals of Dental Specialty* | 2 |
| *Analytical and Quantitative Cytopathology and Histopathology* | 2 |
| *Biomedicines* | 2 |
| *Brazilian Dental Journal* | 2 |
| *Brazilian Oral Research* | 2 |
| *Biomaterials Science* | 2 |
| *BDJ Open* | 2 |
| *Braz Oral Research* | 2 |
| *Clinical Oral Investigations* | 2 |
| *Coatings* | 2 |
| *Child* | 2 |
| *European Journal of Dentistry* | 2 |
| *Ezmialem Science* | 1 |
| *Frontiers in Dentistry* | 1 |
| *Folia Medica Cracoviensa* | 1 |
| *General Dentistry* | 1 |
| *Gerontology* | 1 |
| *Healthcare* | 1 |
| *International Journal of Adhesion and Adhesives* | 1 |
| *International Journal of Periodontics & Restorative Dentistry* | 1 |
| *International Journal of Biomaterials* | 1 |

| | |
|---|---|
| *Journal of Esthetic and Restorative Dentistry* | 1 |
| *Journal International of Pharmaceutical Research* | 1 |
| *Journal of Advanced Oral Research* | 1 |
| *Journal of Adhesive Dentistry* | 1 |
| *Journal of Oral Biology and Craniofacial Research* | 1 |
| *Journal of Evolution of Medical and Dental Sciences* | 1 |
| *Journal of International Oral Health* | 1 |
| *Journal of Pakistan Medical Association* | 1 |
| *Journal of Radiation Research and Applied Sciences* | 1 |
| *Journal of oral Science* | 1 |
| *Journal of Adhesion Science and Technology* | 1 |
| *Journal of Hard Tissue Biology* | 1 |
| *Journal of Conservative Dentistry and Endodontics* | 1 |
| *Meandros Medical and Dental Journal* | 1 |
| *Minerva Stomatologica* | 1 |
| *Medicina* | 1 |
| *Microscopy Research and Technique* | 1 |
| *Nigerian Journal of Clinical Practice* | 1 |
| *Ozone-Science & Engineering* | 1 |
| *Open Dentistry Journal* | 1 |
| *Odontology* | 1 |
| *Odontoestomatologia* | 1 |
| *Oral Surgery, Oral Medicine, Oral Pathology and Oral Radiology* | 1 |
| *Pakistan Journal of Medical & Health Sciences* | 1 |
| *PeerJ Journal* | 1 |
| *Pertanika Journal of Science and Technology* | 1 |
| *Prosthodont Journal* | 1 |
| *Periodica Polytechnica-Mechanical Engineering* | 1 |
| *Restorative Dentistry and Endodontics* | 1 |
| *Royal Society Open Science* | 1 |
| *Romanian Journal of Oral Rehabilitation* | 1 |
| *Science World Journal* | 1 |
| *Učinak onečišćenja slinom na mikropropuštanje otvorenih sendvič-restauracija* | 1 |

**Table S5. List of the first authors that contribute to the included studies.**

| 1st Authors | |
|---|---|
| Abdulhamid Al Ghwainem | Alaaeldin Elraggal |
| Dilber Bilgili Can | Rana Sulaiman Al-Hamdan |
| Rachana Shishodia | Basanagouda S Patil |
| Alpana Kumari | Hyun-Jung Kim |
| Krishnan Venugopal | Seda Nur Karakas |
| Leyla Kerimova | Kopalle Chandra Lekha |
| D Bishayi | Aniket Chavan |
| Diana Bănuț Oneț | Sukhdeep Singh |
| Mahtab Memarpour | Nidhal Salim Dilian |
| Mariana Costa Lima Bandeira | Ramachandra Sujith |
| Rachna Mulani | Samaneh Hesami |
| Deebah Choudhary | Anjali Sardana |
| Poonam Shingare | Nidhi S Thakur |
| Raghavendr Singh | Rani Somani |
| Ricardo Jefferson Pereira | Khushboo Jain |
| M Wendlinger | Ekta Varma Sengar |
| Keyur H Joshi | Ankita Khandelwal |
| Heer Kadhi | Rushikesh Ramesh Mahaparale |
| Arti Dixit | Ziada Saida |
| Gokcen-Deniz Bayrak | Jeswin M Thomas |
| Shlomo Elbahary | Jose Jacob |
| Fahim Vohra | Sarveshwari Singh |

Somayeh Kameli

Vinoo Mathew

Migha Mathew

Namith Rai

Maryam Mohamed ElMansy

Manjari S Gundewar

Marius G Bud

Pratima Shenoi

Amel M Ali

Abdullah Saleh Aljamhan

Maria Salem Ibrahim

Haifa Aldakhil

Suad Soliman Al Khowaiter

Faizan Javed

Satheesh B Haralur

Saira Aslam

Mais Jasim

Asmaa A Mosleh

Geeta Hiremath

B Keskus

Karbasi Kheir

Abisoluwa Adeyeye

Angelo Putignano

Renata Webster Duarte

Aspasia Katsimpali

Prasanti Pradhan

Anju Singh

Sohaib Qais Alwan

Mehmet Guvenc

Azita Kaviani

Ali Nassaj

Dovelin Witty

H A Thomas

Cigdem Celik

Sarah Osama

Nambodri Satish

Natalia Samanta Dutra

Mustafa Khan

Abdul Samad Khan

Marta Nunes Ferreira

Mohammed Bin-Shuwaish

Haiyang Zhang

Meifeng Zhang

Funda Yılmaz

Mohammad Reza Malekipour

Ahmed Heji Albaqawi

Fan Yu

Basak Kiziltan Eliacik

Sukamon Kochotwuttinont

Colette W Stewart

Morakot Piemjai

Madhura Pawar

Yi-Fang Lo

Cansu Atalay

Thrasyvoulos Sfeikos

Neslihan Ozveren

Yanyan Han

Fernanda Manfroi

Marwa Adel

T J Vertolli

Aulfat Albahari

Ebaa Alagha

Nitin Lokhande

V Mubeena

Kaixuan Yan

Snigdha Gavini

Fereshth Alavi

Arya Unnikrishnan

Eby Varghese

Shima Ghasemi

Payel Banerjee

Niveditha Bhupanapadu

Mhd Amer Bashi

Aline Campos Otani

Hamsa Al-Assadi

Hasan Alzoubi

Farahnaz Sharafeddin

A Robaian

Didem Sakaryali

Arpita Deb

Abdul Afraaz

Mohammed Kassim Gholam

Nandini Biradar

Ziad Al-Dwairi

Apra Butail

Arwa Daghrery

Caceres Diaz

Semanur Ozudogru

Elham Zajkani

Suzan Cangul

Naval Aziz

Atiyeh Feiz

Pedro Neves

Tanuja Prabahar

Doaa R M Ahmed

Zaid Al-Jeaidi

Mohammad H Alrefeai

Ali Alrahlah

Shaymaa I Habib

Konstantin Scholz

Thidarat Angwarawong

Nelly Pradelle-Plasse

Esra Ozyurt

Yair Schwimmer

Mewan Salahalddin Abdulrahman

Tarek Elshazly

Xiangqin Xu

C S Sampaio

Tsanka Dikova

Camila Correia

Emese Battancs

Jiahui Zhang

Shelan Jumaah

Yasser M Al-Qahtani

Saurabh Gupta

Hayrunnisa Şimşek

Behnaz Ebadian

Jill C Watson

Shahram Mosharrafian

Maria De Leon Caceres

Andreea Ciurea

Andres Cardenas

Amo Correia

Yasir Alnakib

Sahar Bajabaa

Aylin Cilingir

Halah Alkhawaja

Yueshan Zhou

Sara Valizadeh

Abd-Ellatif El-Patal

Xinyue Ma

Sevda Mihailova Yantcheva

İsmail Hakkı Baltacioğlu

Samah Saker

Zanbaq Hanoon

Hadeer Ibrahim

Saba Tohidkhah

Magrur Kazak

Zeynep Kaynar

Antonio Signore

Zeeshan Qamar

PiYu Hsu

Brandon Peters

Vicenzo Tosco

Niloofar Azimi

Apa Juntavee

Madeeha Bangash

Mahalaskshmi Kumaraguru

Jayashri Prabakar

Dennis Dennis

Kooshan Moradi

César F Cayo-Rojas

Isabel Sinche-Ccahuana

Giancarlo Sarmiento

Jenny López-Torres

Awiruth Klaisiri

P Shankar

Nada Ismail Mohamed

Umer Daood

Pradeep Koppolu

Ghada Naguib

Osvaldo Zmener

Samer Al-Saleh

Yaoxin Wang

Qiuju Li

Nunes Gomes Miranda

Natasha Gupta

Juan Ordonez-Aguilera

Marco Ferrari

Omidi Ranjbar

Chutima Techa-Ungkul

Kianoosh Mirzaei

C Deger

Sura Alani

Razieh Hoseinifar

Mahshid Saffarpour

Elzbieta Zarzecka-Francida

Marianna Barbosa

Mihai-Octavian Boaru

Fabio Rizzante

Lanna Da Costa Vieira

Mariana Pinto

Najmeh Mohammadi

Jelena Juloski

T A Bakhsh

Siriboina Sirisha

Anshula Deshpande

Zhifan Bao

Francesca Zotti

Levente Borhy

Yong Chen

Simona Stoleriu

Xiaowei Guo

Cem Peskersoy

Maryam Shahnavazi

Nojoud Alshehri

Mustafa Yollar

Nazmiye Donmez

Hoda Shirvan

Reveena Benny

Fereshteh Shafiei

Ceyda Akin

Burcu Oglakci

Ellen Scultz-Kornas

Krishna Bharat Shah

F Yu

**Table S6**. Summary of the information retrieved from the 272 results.

| *Microleakage assessment technique* | *Article Title* | *Author & year* | *Type of assessment* | *Sample Type* | *Restoration Type* | *Variables of the Protocol* |
|---|---|---|---|---|---|---|
| ***Colorimetric Method Methylene Blue*** | Promoting Bond Durability by a Novel Fabricated Bioactive Dentin Adhesive | Li *et al.* (2024) | Quantitative<br><br>immersion of the samples in 65% citric acid for 24h + ELISA reader | 3ºmolars | Direct Class I | 0,5% for 24h at 23 ± 2◦C + stereomicroscope |
| | Bond Strength, Microleakage, Microgaps, and Marginal Adaptation of Self-adhesive Resin Composites to Tooth Substrates with and without Preconditioning with Universal Adhesives | *Elraggal et al.* (2024) | Semi-quantitative (MSS) | molars | Direct Class II | 2% for 24h at 37ºC + (Stereomicroscope (20x) |
| | Comparative Assessment of Marginal Micro Leakage of Different Esthetic Restorative Materials Used on Primary Teeth: An In-vitro Study. | Al Ghwainem *et al.* (2024) | Semi-quantitative (MSS) | Primary molars | Direct Class V | 2% for 24h + stereomicroscope |

| | | | | | |
|---|---|---|---|---|---|
| Restorations of Class II Cavities Evaluated for Marginal Leakage When Restored with Composites or a Giomer Using Different Bonding Agents. | Gupta *et al*. (2023) | Qualitative | Molars | Direct Class II | 2% for 24h + stereomicroscope |
| Microleakage, microgap, and shear bond strength of an infiltrant for pit and fissure sealing | Zhou *et al*.(2023) | Semi-quantitative | Molars | Direct Sealant Fissures | 2% for 24h + stereomicroscope (50x) |
| Evaluating the effect of three fissure preparation techniques on microleakage of a colored flowable composite used as a fissure sealant | Eliacik *et al*.(2023) | Semi-quantitative (MSS) | Molars | Direct Sealant Fissures | 2% for 48h + (Stereomicroscope + Digital camera) |
| In Vitro comparison of the physical and mechanical properties of an ormocer with an ormocer-based composite and a nanocomposite restorative material | Karien *et al*. (2023) | Semi-quantitative (MSS) | Permanent Teeth | Direct Class V | 2% for 48h + Stereomicroscope (25x) |

| | | | | | |
|---|---|---|---|---|---|
| Assessment of Embrace-WetBond and Fissurit F Pit and Fissure Sealants' Marginal Sealing Abilities | Joshi *et al*(2023) | Qualitative | Pre-molar | Direct Sealant Fissures | 2% for 24h + stereomicroscope |
| A novel application of a bioactive material as a pit and fissure sealant:In Vitro pilot study evaluating the sealing ability and penetration | Bishayi *et al.* (2023) | Semi-quantitative (MSS) | Permanent teeth | Direct Sealant Fissures | 1% for 24h + Binocular Microscope (25x) |
| The Influence of Chlorhexidine Gluconate Dentine Pre-Treatment on Adhesive Interface and Marginal Sealing | Boaru *et al.*(2023) | Semi-quantitative (MSS) | Molars | Direct Class V | 1% for 24h + Optical Microscope |
| Comparative Evaluation of Microleakage of Two Variables of Glass-Ionomer Cement: An In vitro Study | Saida *et al.* (2023) | Semi-quantitative (MSS) | Molars and premolars | Direct Class II | 1% for 48h + (Stereomicroscope + Digital camera) |
| Investigation of microleakage in adhesive restorations under hyperbaric conditions: An In Vitro study | Ozyurt *et al.* (2023) | Qualitative | Molars | Direct Class II | 1% for 24h + Stereomicrospe |

| | | | | | |
|---|---|---|---|---|---|
| Low shrinkage bulk-filled dental resin composites with non-estrogenic dimethacrylate | Ma *et al*. (2023) | Qualitative | Permanent Teeth | Direct Class V | 2% for 24h + Stereomicroscope |
| Effects of different removal methods of excess resin adhesive on the microleakage of alumina all-ceramic crowns | Zhang *et al.* (2023) | Semi-quantitative (MSS) | 3ºmolars | Indirect | 2% for 24h at 37ºC + stereomicroscope (40x) |
| Microleakage and penetration capability of various pit and fissure sealants upon different sealant application techniques | Juntavee *et al.* (2023) | Quantitative (ImageJ) | premolars | Direct Sealant Fissures | 5% for 24h at 37ºC + (Polarized Light Microscope 40x + Digital camera) |
| Comparison between Different Bulk-Fill and Incremental Composite Materials Used for Class II Restorations in Primary and Permanent Teeth: In Vitro Assessments. | Ibrahim *et al.* (2023) | Semi-quantitative (MSS) | Primary and Permanent teeth | Direct Class II | 1% for 2h + Stereomicroscope |
| Comparison of the marginal microleakage of a bioactive composite resin and | Adeyeye *et al.* (2023) | Semi-quantitative (MSS) | Permanent molars | Direct Class I | 1% for 24h + Digital Microscope |

| | | | | | |
|---|---|---|---|---|---|
| traditional dental restorative materials. | | | | | |
| Effect of the Combination of Restorative Material and the Etching Protocol in Enamel Microleakage in Class II Cavities after Thermocycling | Dutra *et al.* (2023) | Quantitative (ImageJ) | 3ºmolars | Direct Class II | 2% for 48h at 37ºC + Stereomicroscope |
| Microleakage Assessment of Different Preparation Techniques and Pit and Fissure Sealants in Permanent Molars | Bangash *et al.* (2023) | Semi-quantitative (MSS) | Permanent molars | Direct Sealant Fissures | 1% for 24h at 37ºC + Stereomicrospe (20x) |
| Comparative evaluation on the effect of different cavity disinfectant nano gels; Chlorohexidine, Propolis, Liquorice versus Diode Laser in terms of composite microleakage (comparative in vitro study) | ElMansy *et al.* (2023) | Quantitative (Omnimet) | premolars | Direct Class V | 2% for 24h + Stereomicroscope (100x) |
| Evaluation of Self-Adhesive Composite | Bashi *et al.*(2023) | Semi-quantitative (MSS) | Primary canines | Direct Class V | 1% a 24h + Stereomicrospe |

| | | | | | |
|---|---|---|---|---|---|
| Restorations Bond on Primary Canines: An in vitro Study | | | | | |
| Comparative Evaluation of Microleakage With Total-Etch, Universal (Self-Etch Mode), and Nano Adhesive Systems in Class V Composite Restorations: An In-Vitro Study | Singh *et al.* (2023) | Semi-quantitative (MSS) | premolars | Direct Class V | 2% a 24h + Stereomicroscope (10x) |
| Comparative Evaluation of the Microleakage of Cention N and Glass Ionomer Cements in Open-Sandwich Class II Restorations- an in vitro Study | Rai *et al.* (2023) | Semi-quantitative (MSS) | 1ºmolars | Direct Class II | 2% a 72h + stereomicoscope (20x) |
| Physico-Mechanical Characteristics of Ormocer and Bulk Fill Composite Resin Restorative Materials: An in-vitro Study | Sirisha *et al.*(2023) | Semi-quantitative (MSS) | Premolars | Direct Class V | 2% + Stereomicroscope |
| Assessment of microleakage and interfacial adaptation following | Kadhi *et al.* (2023) | Semi-quantitative (MSS) | Primary molars | Direct Class I | 1% a 24h at 37ºC + Stereomicoscope (10x) |

| | | | | | |
|---|---|---|---|---|---|
| simultaneous activation technique in primary molars | | | | | |
| Evaluation of the effect of high-intensity light-curing device on micro-leakage of pits and fissure sealants | Alzoubi *et al*. (2023) | Semi-quantitative (MSS) | Premolars | Direct Sealant Fissures | 1% for 24h + photographed using a Portable LCD Digital Microscope |
| Evaluating the effect of preheating on resin composites in pit-and-fissure caries treatments with a digital intraoral scanner | Yu *et al.* (2023) | Semi-quantitative (MSS) | Human Molars | Direct Class V | 1% for 24h + Optical microscope |
| An In vitro Study of Three Types of Pit and Fissure Sealants for Viscosity, Resin Tag, and Microleakage: A Scanning Electron Microscope Study | Singh *et al*. (2022) | Semi-quantitative (MSS) | premolars | Direct Sealant Fissures | 1% for 24h + Stereomicoscope |
| Comparative Evaluation of Micro Tensile Bond Strength and Microleakage of Ionoseal Glass-Composite as a Fissure Sealant Material, Following Four | Hesami S *et al.* (2022) | Semi-quantitative (MSS) | Human teeth | Direct Sealant Fissures | 5% for 24h at 37ºC + Stereomicrocope (40x) |

| | | | | | |
|---|---|---|---|---|---|
| Different Enamel Surface Pretreatments. | | | | | |
| Comparison of Dentinal Microleakage in Three Interim Dental Restorations: An *In Vitro* Study | Al Khowaiter *et al.* (2022) | Qualitative | Premolars | Direct Class I | 1% for 24h + Digital microscope (50x) |
| Comparison of Microleakage of Composite and Glass Ionomer Restorations in Primary Molars Pretreated with Silver Diamine Fluoride at Two Time Intervals: An In Vitro study | Jasim *et al.* (2022) | Quantitative (Optika Vision lite 2.1 software ) | Primary molars | Direct Class V | 2% for 24h + Stereomicroscope (20x) |
| The Effect of Different Placement Strategies of Bulk Fill Composite on Marginal Leakage of Classii Restoration (Comparative In Vitro Study) | Alani *et al* (2022) | Semi-quantitative (MSS) | Premolars | Direct Class II | 2% for 24h + Digital microscope (40x) |
| Evaluation of Microleakage of Different Types of Pit and Fissure Sealants: An in vitro Comparative Study. | Prabahar *et al* (2022) | Semi-quantitative (MSS) | Premolars | Direct Sealant Fissures | 5% for 24h + Stereomicroscope (40x) |

| | | | | | |
|---|---|---|---|---|---|
| Comparison of microleakage of different surface pre-treatment modalities of ionoseal® pit and fissure sealant in primary teeth: An in vitro study. | Mosleh *et al.*(2022) | Quantitative (ImageJ) | Human Molars | Direct Sealant Fissures | 2% for 24h at 37ºC + Light Microscope |
| Comparative Evaluation of Micro Tensile Bond Strength and Microleakage of Ionoseal Glass-Composite as a Fissure Sealant Material, Following Four Different Enamel Surface Pretreatments | Hesami *et al.* (2022) | Semi-quantitative (MSS) | Premolars | Direct Sealant Fissures | 5% for 24h at 37ºC + Stereomicrocope (40x) |
| Evaluation of micro-CT in the assessment of microleakage under bulk fill composite restorations. | Daghrery *et al.* (2022) | Quantitative (ImageJ) | Human Molars | Direct Class II | 5% for 24h at 25ºC + Optical microscope (20x) |
| Comparative Microleakage Evaluation through the Interfaces between the Tooth and Cement after Stainless Steel Crown Cementation in | Gundewar *et al.* (2022) | Quantitative | Primary molars | Indirect | 1% for 24h + Digital microscope (40x) |

| | | | | | |
|---|---|---|---|---|---|
| Primary Molars: An in vitro Study. | | | | | |
| Marginal microleakage and modified microtensile bond strength of Activa Bioactive, in comparison with conventional restorative materials. | Tohidkhah *et al* (2022) | Quantitative | 3ºmolars | Direct Class II | 2% for 24h + Stereomicroscope (30x) |
| Comparative evaluation of microleakage and hardness of newer posterior restorative materials. | Sardana *et al.* (2022) | Semi-quantitative (MSS) | 3ºmolars | Direct Class II | 2% for 24h at 37ºC + Stereomicroscope (20x) |
| Microleakage Evaluation of Expired and Non-expired Resin Composites and Bonding Agents:In vitro Study | Cilingir *et al.* (2022) | Semi-quantitative (MSS) | Human molars | Direct Class II | 1% for 24h + Stereomicroscope (20x) |
| Fracture Resistance and Microleakage around Direct Restorations in High C-Factor Cavities | Battancs *et al.* (2022) | Semi-quantitative (MSS) | Human molars | Direct Class I | 1% for 24h + Stereomicoscope (40x) |
| A comparative evaluation of | Kumari et al (2022) | Semi-quantitative (MSS) | Premolars | Direct Class V | 0,5% for 24h + stereomicroscope (40x) |

| | | | | | |
|---|---|---|---|---|---|
| microleakage and dentin shear bond strength of three restorative materials. | | | | | |
| Evaluation of Microleakage at the Junction Between Combined Amalgam/Composite Resin Restorations Using Different Bonding Systems in Class II Cavities | Zajkani *et al* (2022) | Semi-quantitative (MSS) | Premolars | Direct Class II | 10% for 24h + stereomicroscope (40x) |
| Effect of Er:YAG laser pretreatment on glass–ceramic surface in vitro | Yan *et al.* (2022) | Quantitative | 3ºmolars | Indirect | 2% for 24h + stereomicroscope |
| An In Vitro Microleakage Study for Comparative Analysis of Two Types of Resin-based Sealants Placed by Using Three Different Types of Techniques of Enamel Preparation | Shingare *et al.* (2021) | Qualitative | Human teeth | Direct Sealant Fissures | 5% for 4h + stereomicroscope (40x) |
| Assessment of Penetration Depth and Microleakage of Different Pit and Fissure Sealants Using | Dixit *et al*. (2021) | Quantitative (ImageJ) | premolars | Direct Sealant Fissures | 1% for 24h + stereomicroscope (10x) |

| | | | | | |
|---|---|---|---|---|---|
| Dye Penetration Method: An In Vitro Study. | | | | | |
| Bond strength and microleakage of different types of cements in stainless steel crown of primary molar teeth | Kameli *et al* (2021) | Quantitative | Primary molars | Indirect | 1% for 24h + stereo-optical-microscope (60x) |
| Comparative Evaluation of Marginal Integrity of Three Esthetic Restorative Materials - An In-vitro Study | Singh *et al.* (2021) | Qualitative | premolars | Direct Class V | 2% for 48h + stereomicroscope (20x) |
| Comparison of Microleakage under Amalgam Restoration: An In Vitro Study. | Hiremath *et al* (2021) | Quantitative (ImageJ) | premolars | Direct Class I | 0,5% for 24h at 37ºC + stereomicroscope (10x) |
| Does Cavity Disinfectant Affect Sealing Ability of Universal Self-etch Adhesive? | Pradhan *et al* (2021) | Semi-quantitative (MSS) | premolars | Direct Class V | 1% for 24h + stereomicroscope (20x) |
| Effect of Different Bonding Techniques on Marginal Adaptation of Harmonize Composite Resin at Different Storage Times | Alagha *et al.* (2021) | Semi-quantitative (MSS) | premolars | Direct Class II | 2% for 24h at 37ºC + stereomicrospe (50x) |

| | | | | | |
|---|---|---|---|---|---|
| Effect of nano-coating on microleakage of different capsulated glass ionomer restoration in primary teeth: An In Vitro study | Alwan *et al.* (2021) | Semi-quantitative (MSS) | Primary molars | Direct Class V | 2% for 24h + stereomicroscpe (40x) |
| Evaluation of microleakage of micro hybrid composite resins versus chitosan-incorporated | Arpita *et al.* (2021) | Quantitative<br><br>immersion in 65% acid nitric + spectrophotometer | premolars | Direct Class V | 2% for 24h |
| Immediate and Long Term Gingival Marginal Leakage of Two Bioactive Bulk Fill Restorative Materials (A Comparative In Vitro Study) | Jumaah *et al* (2022) | Qualitative | premolars | Direct Class II | 2% for 24h + Digital microscope (40x) |
| Impact of graphene oxide and silver diamine fluoride in comparison to photodynamic therapy on bond integrity and microleakage scores of resin modified glass ionomer cement to demineralized dentin | Al-Qahtani *et al.*(2021) | Qualitative | 3ºmolars | Direct Class I | 2% for 24h + Digital microscope |

| | | | | | |
|---|---|---|---|---|---|
| Microleakage in premolar class I restorations between nanohybrid and microhybrid composites: A comparative In Vitro study | Sarmiento *et al*. (2021) | Semi-quantitative (MSS) | premolars | Direct Class I | 2% for 24h + stereomicroscpe (50x) |
| Pit and fissure depth in the enamel of mandibular third molars: An open gate for microleakage? | Zmener *et al.* (2021) | Semi-quantitative (MSS) | 3ºmolars | Direct Sealant Fissures | 1% for 48h at 37ºC + Stereomicroscope (14x) |
| Sealing Ability of Three Different Surface Coating Materials on Conventional and Resin Modified Glass Ionomer Restoration in Primary Anterior Teeth: An In Vitro Study | Deshpande *et al.* (2021) | Semi-quantitative (MSS) | Anterior Primery Teeth | Direct Sealant Fissures | ? for 24h at 37ºC + Stereomicroscope (25x) |
| Study regarding immediate marginal adaptation of a self-adhesive composite resin used for direct restoration | Stoleriu *et al.* (2021) | Semi-quantitative (MSS) | Human Molars | Direct Class V | 2% for 24h + optic microscope (20x) |
| The Effect of Scuba Diving on | Shahnavazi *et al.* (2021) | Semi-quantitative (MSS) | premolars | Direct Class II | 2% for 24h + Stereomicroscope (40x) |

| | | | | | |
|---|---|---|---|---|---|
| Microleakage of a Class II Composite Restoration: An In-Vitro Study | | | | | |
| Effect of Nd:YAG and Er:YAG laser tooth conditioning on the microleakage of self-adhesive resin cement. | Kaviani *et al.* (2021) | Semi-quantitative (MSS) | premolars | Direct Class V | 2% for 24h + Stereomicroscope |
| Marginal quality of a full-body bulk-fill composite placed with an universal adhesive system in etch-and-rinse and self-etch mode: An in vitrostudy. | Signore *et al.* (2021) | Semi-quantitative (MSS) | Human Molars | Direct Class II | 1% for 24h + stereomicroscope (50x) |
| Comparing the Ability of Various Resin-Based Composites and Techniques to Seal Margins in Class-II Cavities. | Aljamhan *et al.* (2021) | Semi-quantitative (MSS) | premolars | Direct Class II | 0,5% for 24h + stereomicroscope (50x) |
| Influence of Nanocoats on the Physicomechanical Properties and Microleakage of Bulk-fill and Resin-modified Glass Ionomer | Habib *et al*. (2021) | Quantitative (ImageJ) | 3ºmolars | Direct Class V | 2% for 24h at 37ºC + stereomicroscope (40x) |

| | | | | | |
|---|---|---|---|---|---|
| Cements: An in vitro Study. | | | | | |
| Influence of Ceramic and Substrate Types on the Microleakage of Aged Porcelain Laminate Veneers. | Alnakib *et al*. (2021) | Quantitative (ImageJ) | premolars | Indirect | 2% for 48h at 37ºC + stereomicroscope |
| Analysis of microleakage and marginal gap presented by new polymeric systems in class V restorations: An in vitro study. | Pereira *et al* (2021) | Semi-quantitative (MSS) | Human Molars | Direct Class V | 1% for 48h + stereomicroscope |
| Comparison of microleakage between different restorative materials to restore marginal gap at crown margin. | Haralur *et al*. (2021) | Semi-quantitative (MSS) | Human Molars | Indirect | 0,1% for 24h + stereomicroscope (30x) |
| Microtensile Bond Strength, Marginal Leakage, and Antibacterial Effect of Bulk Fill Resin Composite with Alkaline Fillers versus Incremental Nanohybrid Composite Resin. | Mohamed *et al.(*2021) | Quantitative (ImageJ) | Human Molars | Direct Class II | 0,1% for 24h + stsreomicroscope (50x) |

| | | | | | |
|---|---|---|---|---|---|
| Evaluation of Class II Restoration Microleakage with Various Restorative Materials: A Comparative in vitro Study | Pawar *et al.* (2021) | Semi-quantitative (MSS) | Human teeth | Direct Class II | 0,5% for 48h + stereomicroscope |
| Marginal Adaptation and Micropermeability of Class II Cavities Restored with Three Different Types of Resin Composites-A Comparative Ten-Month in vitro Study | Yantchev *et al.* (2021) | Semi-quantitative and Quantitative ( KLONK Image Measurement) | Human Molars | Direct Class II | 2% for 12h + stereomicroscope (40x) / samples were coated with gold + SEM |
| Microleakage Evaluation in Class V Cavities Restored with Five Different Resin Composites: In vitro Dye Leakage Study | Bajabaa *et al.* (2021) | Quantitative (ImageJ) | premolars | Direct Class V | 2% for 4h + stereomicrocope (45x) |
| Evaluation of Immediate and Delayed Microleakage of Class V Cavities Restored with Chitosan-incorporated Composite Resins: An in vitro Study. | Deb *et al*. (2021) | Quantitative<br><br>immersion in 65% acid nitric for 3 days centrigugation + spectrophotometer (550 nm) | premolars | Direct Class V | 2% for 24h |

| | | | | | |
|---|---|---|---|---|---|
| Evaluation of the Sealing Ability of Direct versus Direct-Indirect Veneer Techniques: An in vitro Study | Abdulrahman *et al.* (2021) | Semi-quantitative (MSS) | Human teeth | Indirect | 2% for 24h + Stereomicroscope (40x) |
| Microleakage Comparative Evaluation of RMGIC and Alkasite with and without Adhesive System in Class V Cavity: An in vitro Study. | Dennis *et al.* (2021) | Semi-quantitative (MSS) | Human teeth | Direct Class V | 2% for 24h at 37ºC + stereomicroscope (20x) |
| Microleakage of porcelain laminate veneers to tooth surfaces prepared withEr: YAG laser | Abd-Ellatif El-Patal *et al.* (2021) | Semi-quantitative (MSS) | Human Central Incisors | Indirect | 2% for 4h + binocular stereomicrospe |
| An in vitro evaluation of microleakage of resin based composites bonded to chlorhexidine-pretreated dentin by different protocols of a universal adhesive system | Bin-Shuwaish *et al.* (2021) | Semi-quantitative (MSS) | premolars | Direct Class V | 2% for 24h + stereomicroscpe (50x) |
| Effects of different antibacterial disinfectants on | Bin-Shuwaish *et al.* (2021) | Semi-quantitative (MSS) | premolars | Direct Class V | 2% for 24h + stereomicroscpe (50x) |

| | | | | | |
|---|---|---|---|---|---|
| microleakage of bulk-fill composite bonded to different tooth structures | | | | | |
| Evaluation of the marginal microleakage of CAD-CAM compared with conventional interim crowns luted with different types of cement: An in-vitro study | Robaian *et al.* (2021) | Semi-quantitative (MSS) | premolars | Indirect | 2% for 24h + Digital microscope (50x) |
| Evaluation of marginal adaptation at interfaces using composite resin to different setting amalgam filling in class ii cavity preparation | Gholam *et al.* (2021) | Quantitative (ImageJ) | premolars | Direct Class II | 2% for 24h + Stereomicroscope (45x) |
| Influence of Diode Laser for the Treatment of Dentin Hypersensitivity on Microleakage of Cervical Restorations | Ahmed *et al.* (2021) | Semi-quantitative (MSS) | premolars | Direct Class V | 1% for 24h + light microscope (40x) |
| An In vitro Comparative Evaluation of Microleakage of ACP | Mulani *et al*. (2021) | Semi-quantitative (MSS) | premolars | Direct Sealant Fissures | 1% for 24h + Stereomicroscope (10x) |

| | | | | | |
|---|---|---|---|---|---|
| Containing Pit and Fissure Sealant and Moisture Tolerant Pit and Fissure Sealant | | | | | |
| Evaluation of microfiltration in restorations with resin Class I. | Díaz *et al.* (2021) | Quantitative (ImageJ) | Human Molars | Direct Class I | 2% for 24h + Camara Canon |
| An In-vitro Study to Determine the Sealing Ability of Biodentine when used as a Class II Restorative Material | Choudhary *et al.* (2020) | Semi-quantitative (MSS) | Human Molars | Direct Class II | 1% for 24h + stereomicroscope (30x) |
| Cervical Microleakage in Giomer Restorations: An In Vitro Study. | Patil *et al.* (2020) | Semi-quantitative (MSS) | Human Molars | Direct Class II | 2% for 24h at 37ºC + remove the nail varnish + Stereomicrocope (25x) |
| Comparative Evaluation of Compressive Strength, Diametral Tensile Strength, Microhardness, Microleakage, Fluoride Release and Recharge of Resin Modified Gic (Fuji Ii Lc), Bioactive Restorative (Activa Kids) and Alkasite | Lekha *et al.* (2020) | Semi-quantitative (MSS) | Primary molars | Direct Class I | 1% for 24h + stereomicroscope |

| | | | | | |
|---|---|---|---|---|---|
| Restorative (Cention N): An In Vitro Study | | | | | |
| Comparative evaluation of occlusal pits and fissures morphology modification techniques before application of sealants: An In Vitro study | Singh *et. al.* (2020) | Semi-quantitative (MSS) | premolars | Direct Sealant Fissures | 1% for 24h at 37ºC + stereomicroscope (10x) |
| Effect of saliva contamination on microleakage of open sandwich restorations | Çelik *et al.* (2020) | Quantitative (ImageJ) | Human Molars | Direct Class II | 0,5% for 24h + stereomicroscope (20x) |
| Evaluation of Marginal Microleakage and Depth of Penetration of Different Materials Used as Pit and Fissure Sealants: An In Vitro Study. | Butail *et al.* (2020) | Semi-quantitative (MSS) | premolars | Direct Sealant Fissures | 5% for 24h + stereomicroscope (10x) |
| Evaluation of microleakage of temporary crowns made by auto mix and three dimensional | Ghasemi *et al.* (2020) | Semi-quantitative (MSS) | premolars | Indirect | 2% for 24h + stereomicroscope (50x) |

| | | | | | |
|---|---|---|---|---|---|
| printing methods using various types of temporary cements | | | | | |
| Finite Element Analysis in Setting of Fillings of V-Shaped Tooth Defects Made with Glass-Ionomer Cement and Flowable Composite | Dikova *et al*. (2020) | Quantitative | Permanent incisors | Direct Class V | 2% for 8h + optical microscope |
| Hydrolysis-resistant and stress-buffering bifunctional polyurethane adhesive for durable dental composite restoration | Zhang *et al*. (2020) | Semi-quantitative (MSS) | Human Molars | Direct Class V | 1% for 4h at 37ºC + stereomicroscope |
| Laser tooth preparation for pit and fissure sealing | Schwimmer *et al*. (2020) | Quantitative | 3ºmolars | Direct Sealant Fissures | 1% for 24h + stereomicroscope (25x) |
| The influence of a selected desensitizer on marginal microleakage of a class V composite restoration subjected to thermocycles: na in vitro study. | Zarzecka *et al.* (2020) | Quantitative | Molars and premolars | Direct Class V | ? For 6h + digital microscope with a micrometer scale |
| Dimensional Stability and Microleakage of Smart Dental | Katsimpali (2020) | Semi-quantitative (MSS) | Primary carious molars | Carie removal and restoration | 1% for 4h + digital microscope |

| | | | | | |
|---|---|---|---|---|---|
| Composites in Primary Teeth | | | | | |
| Comparison Between Microleakage of Fissure Sealants Placed with or Without Etch and Rinse Adhesive Systems | Aldakhil (2020) | Quantitative (Omnimet) | 3ºmolars | Direct Sealant Fissures | 2% for 24h + optical microscope |
| Microleakage of resin infiltration in artificial white-spot lesions | Klaisiri *et al.* (2020) | Semi-quantitative (MSS) | premolars | Formation of artificial white-spot lesions, removal and restoration | 2% for 24h at 37ºC + stereomicrospe (40x) |
| Influence of fractional carbon-dioxide laser in comparison to ErCr-YSGG on the dentin bond integrity of bioactive materials. | Al-Jeaidi *et al.* (2020) | Quantitative (ImageJ) | Permanent Teeth | Not mention | 2% for 24h + digital microscope (Hirox) |
| Evaluation of Marginal Adaptation of Composite Restorations Reinforced with Novel Enamel Inserts (Biofillers) in Class V Cavities. | Biradar *et al.* (2020) | Semi-quantitative (MSS) | premolars | Direct Class V | 1% for 4h + stereomicroscope (10x) |

| | | | | | |
|---|---|---|---|---|---|
| Comparative Evaluation of Microleakage in Cavities Restored with Nanohybrid and Microfilled Composites Using Oblique Incremental Technique- An in vitro - Study | Shah *et al*. (2020) | Semi-quantitative (MSS) | Human teeth | Direct Class I | 2% for 24h + stereomicorscope (12x) |
| Evaluation of Two Resin Composites Having Different Matrix Compositions | Elshazly *et al*. (2020) | Semi-quantitative (MSS) | Human Molars | Direct Class V | 1% for 24h + stereomicroscope (15x) |
| Comparative Evaluation of Microleakage of Three Different Generation Dentin Bonding Agents and Resin Modified Glass Ionomer Cement: An In-Vitro Study | Mahaparale *et al*. (2020) | Semi-quantitative (MSS) | premolars | Direct Class V | 0,5% for 24h at 37ºC + Stereomicroscope (20x) |
| Does etching time affect the in vitro performance of a sealant material? | Lo *et al*. (2020) | Quantitative (imageJ) | premolars | Direct Sealant Fissures | 0,5% for 24h at 37ºC + Stereomicroscope (20x) |
| In Vitro Evaluation of Different Protocols for Preventing Microleakage of | Şimşek *et al*. (2020) | Quantitative (ImageJ) | premolars | Direct Sealant Fissures | 0,5% for 24h at 37ºC + Stereomicroscope (20x) |

| | | | | | |
|---|---|---|---|---|---|
| Fissure Sealants Placed Following Saliva Contamination | | | | | |
| Evaluation of the effect of different light cure devices/modes on the micro-leakage of class V composite restoration: (A comparative in vitro study) | Al-Assadi *et al.* (2020) | Semi-quantitative (MSS) | premolars | Direct Class V | 2% for 24h + stereomicroscope (40x) |
| Effect of Antioxidants in Reducing Microleakage of Composite Restoration in Intracoronally-Bleached Teeth" – An In-Vitro Study | Kumar *et al.* (2020) | Semi-quantitative (MSS) | premolars | Direct Class V | 1% for ? + stereomicroscope |
| Comparative Evaluation of the Effect of Refrigeration on Microleakage of 3 Different Posterior Composites - An In Vitro Study | Mathew *et al.* (2020) | Semi-quantitative (MSS) | Primary molars | Direct Class II | 0,5% for ? + stereomicroscope |
| Evaluation of Microleakage, Shear Bond Strength and Resin Tag Formation Following the Use of Natural Collagen | Banerjee (2020) | Semi-quantitative (MSS) | Primary Teeth | Direct Class V | 2% for ? + stereomicroscope |

| | | | | | | |
|---|---|---|---|---|---|---|
| | Cross Linker, Grape Seed Extract on Primary Molars: Na in Vitro Study | | | | | |
| | Synthesis, characterization, and aging resistance of the polyurethane dimethacrylate layer for dental restorations. | Guo *et al.* (2020) | Quantitative | Human Molars | Direct Class V | 1% for 12h + stereomicroscope (35x) |
| | | | | | | |
| | | | | | | |
| ***Basic Fuchsin*** | Evaluation of the ceramic laminate veneer-tooth interface after different resin cement excess removal techniques. | Otani *et al*. (2024) | Quantitative | Anterior Human Teeth | Indirect | 2% for 24h + Optical Microscope (10) |
| | Effect of protective coating agents on microleakage and flexural strength of glass ionomer cement and zirconomer. an in vitro study. | Thomas *et al*.(2024) | Semi-quantitative (MSS) | Human Teeth | Direct Class V | 2% for 24h + Stereomicroscope |
| | Effect of varying functional monomers in experimental self-adhesive composites: polymerization kinetics, cell | Ferreira *et al.* (2023) | Semi-quantitative (MSS) | Human Molars | Direct Class V | 0,5% a 24h + Stereomicroscope |

| | | | | | |
|---|---|---|---|---|---|
| metabolism influence and sealing ability | | | | | |
| Nano-cellulose Reinforced Glass Ionomer Restorations: Na In vitro study | Mohammadi *et al.* (2023) | Semi-quantitative (MSS) | Primary Molar | Direct Class V | 2% a 24h at 37ºC + Stereomicroscope (30x) |
| Exploration and preliminary clinical investigation of an adhesive approach for primary tooth restoration | Xu *et al.* (2021) | Semi-quantitative (MSS) | Primary Teeth | Direct Class II | 0,5% a 24h + Stereomicroscope (40x) |
| Effect of nano-filled protective coating on the microleakage resistance of a nanocomposite during bleaching treatment | Guvenc *et al.* (2023) | Semi-quantitative (MSS) | Human Teeth | Direct Class V | 0,5% a 24h at 37ºC + Stereomicroscope (40x) |
| The efficacy of lining materials in the reduction of microleakage in class II composite resin restoration using the sandwich technique: A stereomicroscopic study. | Benny *et al.* (2023) | Semi-quantitative (MSS) | premolars | Direct Class II | 0,5% a 24h at 37ºC + Stereomicroscope (40x) |
| Comparative Evaluation of Microleakage in Hall's | Thakur *et. al.* (2023) | Semi-quantitative (MSS) | Primary Molar | Indirect | 0,5% a 24h + Stereomicroscope |

| | | | | | |
|---|---|---|---|---|---|
| with SDF, Hall's, and Conventional Technique Using Different Luting Cements. | | | | | |
| Evaluation of the microleakage of class V composite restoration after cavity treatment with Erbium, CO(2) lasers, Papain, and Bromelain enzymes. | Sharafeddin *et al.* (2023) | Semi-quantitative (MSS) | Human Teeth | Direct Class V | 2% a 24h + Stereomicroscope (40x) |
| Microleakage and Marginal Integrity of Surface-Coated and Laser-Pretreated Class V Composite Restorations in Primary Teeth. | Azimi *et al.* (2023) | Semi-quantitative (MSS) | Primary Molar | Direct Class V | 2% a 24h + Stereomicroscope (40x) |
| Evaluation of Microleakage of Stainless Steel Crowns and Pedo Jacket Crowns after Cementation With Different Luting Cements | Varghese *et al.*(2023) | Quantitative | Primary Teeth | Indirect | 2% for 7 days at 37ºC + Stereomicoscope (50x) |
| The effects of dental adhesives total etch; self-etch and selective | Yollar *et al.* (2023) | Semi-quantitative (MSS) | Human Molars | Direct Class II | 0,5% for 24h + stereomicoscope (100x) |

| | | | | | |
|---|---|---|---|---|---|
| etch application procedures on microleakage in class II composite restorations | | | | | |
| Effect of Prolonged Water Aging on the Bond Strength and Marginal Seal of Three Novel Restorative Materials. | Witty *et al*. (2023) | Quantitative (ImageJ) | Human Teeth | Direct Class V | 0,5% for 24h + stereomicoscope (20x) |
| Evaluation of the Effect of Nanographene Oxide on Microleakage of Conventional and Resin-Modified Glass Ionomer | Sharafeddin *et al*. (2023) | Qualitative | Human Molars | Direct Class V | 2% for 24h + Stereomicroscope (40x) |
| Micro Tensile bond strength and microleakage assessment of total-etch and self-etch adhesive bonded to carious affected dentin disinfected with Chlorhexidine, Curcumin, and Malachite green | Qamar *et al.* (2023) | Semi-quantitative (MSS) | Carious Human Teeth | Carie removal and restoration | ? For 8h |

| | | | | | |
|---|---|---|---|---|---|
| Microleakage Evaluation of Two Methacrylate-Based Composites (GC Kalore and Luna SDI) in Class II Restorations: A Laboratory Study. | Moradi *et al.* (2022) | Semi-quantitative (MSS) | premolars | Direct Class II | 2% for 24h + Stereomicoscope |
| Comparative evaluation of microleakage of various restorative materials in pulpotomized primary molars | Thomas *et. al.*(2022) | Semi-quantitative (MSS) | Primary Molar | Direct Class II | 0,5% for 24h + Stereomicroscope (20x) |
| MARGINAL MICROLEAKAGE OF GLASS IONOMER BASED RESTORATIONS AFTER CONVENTIONAL CAVITY PREPARATION AND ER: YAG LASER IRRADIATION | Kaynar *et al.* (2022) | Semi-quantitative (MSS) | Human Teeth | Direct Class V | 0,5% for 24h + Stereomicroscope (40x) |
| Shear Bond Strength and Microleakage of Pit and Fissure Sealants Placed after | Bao *et al.* (2022) | Semi-quantitative (MSS) | Human Teeth | Direct Sealant Fissures | 0,5% for 24h at 37ºC + Stereomicroscope (20x) |

| | | | | | |
|---|---|---|---|---|---|
| Saliva-Contaminated Etched Enamel | | | | | |
| The Investigation of the Marginal Microleakage of Ceramic Veneer Crowns with Different Finish Lines | Akin *et al*. (2022) | Semi-quantitative (MSS) | premolars | Indirect | 0,5% for 24h at 37ºC + Stereomicroscope (20x) |
| Comparative microleakage outcomes of different techniques used for creating the occlusal anatomy in occlusal direct restorations using the dental operating microscope. | Bud *et al*. (2022) | Semi-quantitative (MSS) | Human Molars | Direct Class II | 0,5% for 24h, unexposed to light + Optical Microscope (20x) |
| Effects of Rinsing Water Temperature and Preheated Composites on Microleakage of Composite Restorations with Two Bonding Agents. | Malekipour *et al*. (2022) | Semi-quantitative (MSS) | Human Teeth | Direct Class V | 0,5% for 24h + Stereomicroscope (32x) |
| Evaluation of micro-CT in the assessment of microleakage under bulk fill composite restorations. | Daghrery *et al*. (2022) | Quantitative (ImageJ) | Human Molars | Direct Class II | 0,5% for 24h at 25ºC + Optical microscope (20x) |

| | | | | | |
|---|---|---|---|---|---|
| Comparison of Microleakage of Composite and Glass Ionomer Restorations in Primary Molars Pretreated with Silver Diamine Fluoride at Two Time Intervals: Na In Vitro study | Jasim *et al.* (2022) | Semi-quantitative (MSS) | Primary Molar | Direct Class V | 0,5% for 24 at 37ºC + Stereomicroscope |
| Efficacy of chlorhexidine, photosensitizers, green tea extract, and propolis on bond integrity and microleakage of caries-affected dentin: An in-vitro study | Albaqawi *et al.* (2022) | Semi-quantitative (MSS) | Carious Human Teeth | Carie removal and restoration | ? For 8h |
| Comparison of physical and mechanical properties of three different restorative materials in primary teeth: an in vitro study | Keskus *et al.* (2022) | Semi-quantitative (MSS) | Human Teeth | Direct Class V | 0,5% for 24 at 37ºC + Stereomicroscope |
| The in vitro effect of antimicrobial photodynamic therapy with toluidine blue and indocyanine green | Saffarpour *et al.* (2022) | Semi-quantitative (MSS) | premolars | Direct Class V | 0,5% for 24 at 37ºC + Stereomicroscope (40x) |

| | | | | | |
|---|---|---|---|---|---|
| on microleakage of class V cavities. | | | | | |
| Noncarious cervical lesion pretreated using antimicrobial photodynamic therapy and diode laser in reducing dentin hypersensitivity bonded to different restorative material: Valuation of bond values and invitro dye leakage | Koppolu *et al.* (2022) | Semi-quantitative (MSS) | premolars | Direct Class V | 0,5% for 24h at 37ºC + Digital microsocpe (40x) |
| Evaluation of microleakage and fatigue behaviour of several fiber application techniques in composite restorations | Ozudogru *et al.* (2022) | Semi-quantitative (MSS) | Human Molars | Direct Class II | 0,5% for 24h in the dark + stereomicroscope |
| Influence of methylene blue, Riboflavin, and indocyanine green on the bond strength of caries affected dentin when bonded to resin-modified glass ionomer cement | Alrefea *et al*. (2022) | Semi-quantitative (MSS) | Carious Human Teeth | Carie removal and restoration | 0,5% for 24h at 37ºC + digital microscope (40x) |

| | | | | | |
|---|---|---|---|---|---|
| Comparative evaluation of microleakage of zinc phosphate cement, resin-modified glass ionomer, and two dual-cure resin cements:in vitro study | Jacob *et. al* (2022) | Semi-quantitative (MSS) | Human Molars | Indirect | 0,5% for 24h + stereomicroscope |
| Adhesion of Flowable Resin Composites in Simulated Wedge-Shaped Cervical Lesions: An In Vitro Pilot Study | Banut *et al*. (2021) | Quantitative (QuickPhoto Micro 2.2 software) | Human Teeth | Direct Class V | 0,5% for 24h + Inverted microscope (40x) |
| In Vitro Study on the Adhesive Performance of Some Resin-Based Materials Used to Restore Class II Cavities | Ciurea *et al*. (2021) | Quantitative (QuickPhoto Micro 2.2 software) | Human Teeth | Direct Class II | 0,5% for 24h + Inverted microscope (40x) |
| The Effects of Thermo-mechanical Aging on Microleakage in Composite Restorations Polymerized Using One New Generation and Two Conventional Led Light Curing Units | Donmez *et al*. (2021) | Semi-quantitative (MSS) | premolars | Direct Class V | 0,5% for 24h + stereomicroscope (40x) |

| | | | | | |
|---|---|---|---|---|---|
| The influence of the operator's experience on the microleakage of two universal adhesives | Shafiei *et al* (2021) | Quantitative (QuickPhoto Micro 2.2 software) | Human Molars | Direct Class V | 0,5% for 24h + digital microscope |
| Does laser etching have an effect on application mode of a universal adhesive?-A microleakage and scanning electron microscopy evaluation. | Atalay *et al* (2021) | Semi-quantitative (MSS) | Human Molars | Direct Class V | 0,5% for 24h + light microscope (40x) |
| In vitro Evaluation of the Effect of Different Luting Cements and Tooth Preparation Angle on the Microleakage of Zirconia Crowns. | Ebadian *et al.* (2021) | Semi-quantitative (MSS) | premolars | Indirect | 0,5% for 48h at 37ºC + stereomicroscope (20x) |
| Evaluation of microleakage in Class II composite restorations: Bonded-base and bulk-fill techniques | Feiz *et al.* (2021) | Semi-quantitative (MSS) | Human Molars | Direct Class II | 0,5% for 24h + stereomicroscope (32x) |
| Effects of magnetic resonance imaging on the microleakage of five restorative | Yilmaz *et al*. (2021) | Semi-quantitative (MSS) | Human Teeth | Direct Class V | 0,5% for 24h at 37ºC + stereomicroscope (10x) |

| | | | | | |
|---|---|---|---|---|---|
| materials: na In Vitro study | | | | | |
| Caries effected dentin disinfection using Ozone, methylthioninium chloride and turmeric activated by photodynamic therapy on bond integrity of resin-modified glass ionomer cement. | Al-Hamdan et al. (2021) | Semi-quantitative (MSS) | Carious Human Teeth | Carie removal and restoration | 0,5% for 24h at 37ºC + microscope (40x) |
| Adhesion of three types of fissure sealant in saliva-contaminated and noncontaminated conditions: an in vitro study. | Memarpour et al (2021) | Quantitative | 3ºmolar | Direct Sealant Fissures | 0,5% for 24h + digital microscope (50x) |
| The effect of dentin age on the microshear bond strength and microleakage of glass-ionomer cements. | Techa-Ungkul et al (2021) | Semi-quantitative (MSS) | Human Teeth | Direct Class V | 0,5% for 24h + The superficial dye was removed with a pumice slurry and rubber + streomicroscope (30x) |
| Influence of methylthioninium chloride, curcumin and Er, Cr: YSGG on caries affected dentin bonded to bioactive and conventional bulk | Alrahlah et al (2021) | Qualitative | Carious Human Teeth | Carie removal and restoration | ? % for 8h + optical microscope (40x) |

| | | | | | |
|---|---|---|---|---|---|
| fill dental restorative material | | | | | |
| A comparison of cuspal movement of premolar teeth restored with bulk-filled composite resins combined with universal adhesives | Kerimova et al (2020) | Semi-quantitative (MSS) | premolars | Direct Class II | 0,5% for 24h + Stereomicroscope |
| Assessment of shear bond strength and microleakage of fissure sealant following enamel deproteinization: An In Vitro study. | Bayrak et al. (2020) | Semi-quantitative (MSS) | 3ºmolar | Direct Sealant Fissures | 0,5% for 24h at 37ºC + stereomicroscope (20x) |
| Comparative evaluation of the effect of artificial aging on the marginal leakage of cast crowns luted with three cements: An In vitro study | Mathew et. Al (2020) | Semi-quantitative (MSS) | premolars | Indirect | 0,5% for 24h + Optical vision microscope |
| Effect of photodynamic therapy on microleakage of class V composite restorations in primary teeth | Nassaj et al (2020) | Semi-quantitative (MSS) | Primary Teeth | Direct Class V | 0,5% for 24h + stereomicroscope |

| | | | | | |
|---|---|---|---|---|---|
| Effect of Silver Diamine Fluoride Pretreatment on Microtensile Bond Strength of Dentin and on Microleakage of Resin Composite in Primary Molars: “An In Vitro Study | Satish (2020) | Semi-quantitative (MSS) | Carious Primary Molar | Carie removal and restoration | 0,5% for 24h + stereomicrocope |
| Evaluation of Caries-Free Restorations Bonded with Various Adhesive Systems: In Vitro Study | Piemjai et al (2020) | Quantitative (Image Pro Plus) | Human Molars | Direct Class V | 0,5% for 24h + stereomicrocope |
| In Vitro Evaluation of Different Protocols for Preventing Microleakage of Fissure Sealants Placed Following Saliva Contamination | Şimşek et al (2020) | Quantitative (ImageJ) | 3ºmolar | Direct Sealant Fissures | 0,5% for 24h at 37ºC + stereomicroscope (20x) |
| In Vitro study of the relationship between bond strength to dental enamel and microleakage in photopolymerizable composite resins | Caceres et al (2020) | Semi-quantitative (MSS) | 3ºmolar | Direct Class V | 0,5% for 24h + Stereomicroscope |
| Microleakage of a Self-adhesive Composite of Class V Cavities: Effect | Valizadeh et al (2020) | Semi-quantitative (MSS) | Human Teeth | Direct Class V | 2% for 24h at 37ºC + stereomicroscope (40x) |

| | | | | | |
|---|---|---|---|---|---|
| of Surface Treatment and Thermocycling. | | | | | |
| The Effect of Occlusal Loading on Gingival Microleakage of Bulk Fill Composites Compared with a Conventional Composite. | Hoseinifar et al (2020) | Semi-quantitative (MSS) | premolars | Direct Class II | 0,5% for 48h + stereomicroscope (40x) |
| Microleakage patterns of glass ionomer cement at cement-band and cement-enamel interfaces in primary teeth. | Shankar et al (2020) | Quantitative (Eletronic Digital caliper) | Primary Molar | Not mention | 0,5% for 24h + stereomicrospe (10x) |
| Marginal Microleakage of Composite Resin Materials Comprising Different Photo Initiators with Surface Sealants and Bonding Agent Application after Thermomechanical Aging | Kazak et al (2020) | Semi-quantitative (MSS) | 3ºmolar | Direct Class II | 0,5% for 24h at 37ºC + stereomicroscope (40x) |
| Evaluation of Caries-Free Restorations Bonded with Various | Piemjai et al (2020) | Quantitative (Image Pro Plus) | Human Molars | Direct Class V | 0,5% for 24h + stereomicrospe (10x) |

| | | | | | |
|---|---|---|---|---|---|
| Adhesive Systems: In vitro Study | | | | | |
| Evaluation of Microleakage Caused by Different LED Light Sources in Composite Resins with AutoCAD Program | Cangul et al (2020) | Quantitative (Auto CAD 2014 software ) | Human Teeth | Direct Class I | 0,5% for 24h + stereomicroscope (40x) |
| Comparison of cuspal movement of premolar teeth restored with bulk-filled composite resins combined with universal adhesives. | Kerimova et al (2020) | Qualitative | premolars | Not mention | 0,5% for 24h + stereomicroscope |
| Comparative Evaluation of Mechanical and Microleakage Properties of Cention-N, Composite, and Glass Ionomer Cement Restorative Materials. | Sujith et al. (2020) | Qualitative | premolars | Direct Class V | 0,5% for 24h + stereomicroscope |
| Comparative Evaluation of Compressive Strength, Microleakage, Fluoride Release and Recharge Ability of Various Glass Ionomer Based Restorative | Basheer (2020) | Qualitative | Primary Teeth | Direct Class I | 0,5% for 24h + stereomicroscope |

| | | | | | | |
|---|---|---|---|---|---|---|
| | Materials: An In Vitro Study | | | | | |
| | | | | | | |
| ***Silver Nitrate*** | In vitro Microleakage and Fracture Resistance of "Infinity Edge" and Cusp Reduction Preparation Designs for Moderate-sized Class II Composites. | Watson et al (2024) | Quantitative (Micro-CT) | Human Molars | Direct Class II | 1º Micro-CT. 2º 50% ammonium Silver Nitrate por 12h + photodevoloping solution with fluorescent light for 8h + Micro-CT |
| | Marginal adaptation of bulk-fill resin composites with different viscosities in class II restorations: a micro-CT evaluation. | Baltacioğlu et al (2024) | Quantitative (Micro-CT) | Human Molars | Direct Class II | 1/1 $AgNO_3$ por 24h in the dark + photodevoloping solution with fluorescent light for 8h + Micro-CT |
| | Polyvinylpyrrolidone as a primer for resin-dentin bonding | Wang et al (2024 | Quantitative (ImageJ) | Human Teeth | Direct Class I | Immersed in 50 wt% (w/v) ammoniacal $AgNO_3$ solution for 24 h. in the dark. + photodevoloping solution with fluorescent light for 8h + Polishing and ultrasonically cleaning + SEM evaluation |
| | Microleakage Assessment of Hydrophilic Pit and Fissure Sealants Fortified with Green Synthesized Silver | Kumaraguru et al (2023) | Qualitative | Human Teeth | Direct Sealant Fissures | 0,1% $AgNO_3$ for 24h + photodevoloping solution with fluorescent light for 12 + stereomicoscope |

| | | | | | |
|---|---|---|---|---|---|
| Nanoparticles- An In Vitro Study | | | | | |
| In Vitro microleakage at the enamel and dentin margins of class II cavities of primary molars restored with a bulk-fill and a conventional composite | Mosharrafian et al (2023) | Quantitative | Primary Molars | Direct Class II | 1M AgNo3 for 6h + photodevoloping solution with fluorescent light for 12h + Stereomicroscope (10x) |
| Cuspal Deflection and Marginal Integrity of Class II Cavities Restored with Bulk-fill Resin Composites | Duarte et al (2023) | Semi-quantitative (MSS) | premolars | Direct Class II | 50% ammonical Silver Nitrate in the dark por 24h + photodevoloping solution with fluorescent light for 16h + Stereomicroscope (25x) |
| Comparing nanoleakage between class II bulkfill and incremental composite restorations using snowplow technique | Ali et al (2023) | Quantitative (Energy Dispersive Analytical X-ray Spectometry ) | Primary and permanent molars | Direct Class II | 50% AgNO3 for 24h in the dark + photodevoloping solution with fluorescent light for 8h + All specimens were coated with gold in a sputter-coating unit + SEM |
| Microleakage in indirect onlay restorations cemented with three different types of adhesives: An In Vitro study. | Sinche-Ccahuana et al (2023) | Semi-quantitative (MSS) | premolars | Indirect | 1M AgNo3 for 24h in the dark + photoreflective solution under fluorescent light for 8 hours + Stereomicoscope (20x |
| In Vitro Microleakage at the enamel and dentin margins of | Mosharrafian et al (2023) | Quantitative | Primary Molars | Direct Class II | 1M AgNO3 for 6h in the dark + photodevoloping solution with |

| | | | | | |
|---|---|---|---|---|---|
| class II cavities of primary molars restored with a bulk-fill and a conventional composite. | | | | | fluorescent light for 12h + Stereomicoscope (10x) |
| 3D-2D microleakage assessment of preheated bulk-fill composite resin applied with different parameters: a micro-CT analysis | Bilgili et al. (2023) | Quantitative (CTAn software) | 3ºmolars | Direct Class II | 50% $AgNO_3$ for 24h + developer solution for 8h + ultrasonicaly cleaned to remove silver particles form the tooth surface + Micro-CT |
| Are universal adhesives in etch-and-rinse mode better than old 2-step etch-and-rinse adhesives? One-year evaluation of bonding properties to dentin. | Wendlinger et al (2023) | Quantitative (ImageJ) | Human Molars | Direct Class I | 50% ammonical Silver Nitrate por 24h + photodevoloping solution with fluorescent light for 8h + Specimens were mounted on aluminum stubs, polished.Then, they were ultrasonically cleaned, air dried and gold sputter coated + SEM evaluation |
| Microleakage of Class II Bulk-Fill Resin Composite Restorations Cured with Light-Emitting Diode versus Quartz Tungsten-Halogen Light: Na in vitro Study in Human Teeth | López-Torres et al (2023) | Qualitative | Human Molars | Direct Class II | 1M $AgNo_3$ for 24h + photoreflective solution under fluorescent light for 8 hours + Stereomicoscope |

| | | | | | |
|---|---|---|---|---|---|
| The effect of dentin surface pretreatment using dimethyl sulfoxide on the bond strength of a universal bonding agent to dentin | Mirzaei et al (2023) | Quantitative (Photophop Software) | 3ºmolars | Direct Class I | 1M AgNo3 for 6h + photodevoloping solution with fluorescent light for 8h + Stereomicroscope |
| Effect of cervical margin relocation on marginal adaptation and microleakage of indirect ceramic restorations | Adel et al (2023) | Semi-quantitative (MSS) | 1ºmolars | Direct Class II | 50% Ammoniacal AgNO3 for 24h. + Nail varnish was removed and each tooth was placed in a test tube with 1:10 diluted photo developer solution for 8 h. + The teeth were sliced using a low-speed water-cooled diamond saw + slices were examined with a digital microscope (35x) |
| The Effect of Dental Adhesive Composition and Etching Mode on Microleakage of Bonding Agents in Primary Molar Teeth. | Ranjbar et al (2022) | Quantitative | Primary Molars | Direct Class II | 1M AgNO3 for 6 h + developer solution for 12 hours, followed by exposure to fluorescent light for 6 hours + Stereomicroscope (10x) |
| Evaluation of micro-CT in the assessment of microleakage under bulk fill composite restorations. | Daghrery et al (2022) | Quantitative (Micro-CT) | Human Molars | Direct Class II | 50% AgNO3 for 24h at 25ºC + photo-developer solution under fluorescent light for 6h + Micro-CT |
| The effects of amalgam contamination and different surface modifications on | Alshehri et al (2022) | Semi-quantitative (MSS) | Human Teeth | Direct Class II | 50% ammoniacal AgNO3 for 24h in the dark + photo-developer solution under fluorescent light for 12h + Digital microscope (50x) |

| | | | | | |
|---|---|---|---|---|---|
| microleakage of dentin bonded to bulk fill composite when using different adhesive protocols. | | | | | |
| Comparative Evaluation of Marginal Microleakage Between Bulk-Fill, Preheated Bulk-Fill, and Bulk-Fill Flowable Composite Resins Above and Below Cemento-Enamel Junction Using Micro-Computed Tomography: An in vitro Study | Dilian et al. (2021) | Quantitative (Micro-CT) | premolars | Direct Class II | 50% ammoniacal AgNO3 for 24h in the dark + photo-developer solution for 8h + Micro-CT |
| Evaluation of Nanoleakage Depth and Pattern of Cervical Restorations Bonded with Different Adhesive Systems. | Bhupanapadu et al (2022) | Quantitative | premolars | Direct Class V | 50% AgNO3 for 24h in total darkness + Immersion in 10% EDTA for 5s to remove the semar layer + SEM |
| Effect of a fiber-reinforcing technique for direct composite restorations of structurally compromised teeth on | Sfeikos et al (2022) | Quantitative (EDS + MSS) | Human Molars | Direct Class I | 50% AgNO3 for 4h + immersed in a developer solution and fluorecent light for 12h + section of the samples + SEM |

| | | | | | |
|---|---|---|---|---|---|
| marginal microleakage. | | | | | |
| Influence of Application of Dimethyl Sulfoxide on the Bonding Properties to Eroded Dentin. | Cardenas et al (2021) | Quantitative (ImageJ) | 3ºmolars | Direct Class I | 50% ammoniacal AgNO3 for 24h + photodeveloping solution for 8 h under fluorescent light + SEM |
| Interface between calcium silicate cement and adhesive systems according to adhesive families and cement maturation | Pradelle-Plasse et al (2021) | Quantitative | Human Molars | Direct Class II | 50% AgNO3 for 12h in the dark + photodeveloping solution for 2h under fluorescent light + Acetone to dissolve the nail varnish + Binocular Loop conected to a cam and analysed using Leica Software + SEM |
| Polymer-Based Bioactive Luting Agents for Cementation of All-Ceramic Crowns: An SEM, EDX, Microleakage, Fracture Strength, and Color Stability Study. | Al-Saleh et al (2021) | Quantitative (Micro-CT) | premolars | Indirect | 50% AgNO3 for 12h in the dark + photodevoloing solution under fluorecent light foor 12h + Micro-CT |
| Microleakage in class II restorations of two bulk fill resin composites and a conventional nanohybrid resin composite: an in vitro | Cayo-Rojas et al (2021) | Semi-quantitative (MSS) | Human Molars | Direct Class II | 1M AgNO3 for 24h in the dark + photodevoloing solution under fluorecent light foor 8h + Stereomicroscope (16x) |

| | | | | | |
|---|---|---|---|---|---|
| study at 10,000 thermocycles. | | | | | |
| Comparison of the Ability of Two Brands of CBCT with That of SEM to Detect the Marginal Leakage of Class V Composite Resin Restorations. | Karbasi et al (2021) | Semi-quantitative (MSS) | premolars | Direct Class V | 50% AgNO3 for 24h in the dark + photodevoloping solution for 8h + CBCT + SEM |
| Nondestructive evaluation of microleakage in restored primary teeth using CP-OCT. | Bakhsh et al (2021) | Quantitative (ImageJ) | Primary Molars | Direct Class V | ? AgNO3 for 24h + photodevoloing solution under fluorecent light foor 8h + CP-OCT system |
| Sealing ability of new translucent zirconia crowns made with digital workflow and cemented with different types of cement | Ferrari et al (2021) | Quantitative | Anterior Teeth | Indirect | 1. 50% AgNO3 for 24h + photodevoloping solution ? + sectioning of the samples + Eclipse microscope<br>2. Sample analysis was completed using SEM in at least one section from each sample at different magnifications. |
| Effect of Cariogenic Challenge on the Degradation of Adhesive-Dentin Interfaces | Manfroi et al (2020) | Quantitative (ImageJ) | 3ºmolars | Not mention | 50% AgNO3 for 24h + photodevoloping solution under fluorescent ligh 8h + 2.5% glutaraldehyde for 12h + distilled water for 1 min + dehydration with rising ethanol concentrations + immerged in 50% HMDS and 50% alcohol at 100% for 5 min + then placed in 100% HMDS for 10 min + SEM + Image J |

| | | | | | |
|---|---|---|---|---|---|
| Evaluating the failure of resin-based materials on the proximal cervical dentin | Kochotwuttinont et al (2020) | Quantitative (ImageJ) | premolars | Direct Class II | 50% ammoniacal AgNO3 for 24h in the dark + photodevoloping solution for 8h under fluorescent light +2,5% glutaraldehyde for 12h + SEM (1000x) |
| Influence of selective caries excavation on marginal penetration of class II composite restorations in vitro | Scholz et al (2020) | Quantitative (Optimas 6.51 software) | premolars | Direct Class II | 50% AgNO3 for 2h in the dark + photodevoloping solution for 6h under fluorescent light + Standardized images were taken from both sides of the sections using a photomacroscope |
| No correlation between two methodological approaches applied to evaluate cervical margin relocation | Juloski et al (2020) | Quantitative | Human Molars | Indirect | 50% ammoniacal AgNO3 for 24h + nail varnish was removed with acetone + Digital Microscope + SEM for assessment of the marginal quality |
| Bond integrity and microleakage of dentin-bonded crowns cemented with bioactive cement in comparison to resin cements:in vitro study. | Vohra et al (2020) | Quantitative (ImageJ) | Human Molars | Indirect | 50% AgNO3 for 12h in the dark + photodevoloping solution for 12h under fluorescent light + Micro-CT |
| Adhesive resistance of a copaiba oil-based dentin biomodifier. | Bandeira et al (2020) | Semi-quantitative (MSS) | Human Molars | Direct Class V | 50% AgNO3 for 2h in the dark + photodevoloping solution for 16h under fluorescent light + Stereomicroscope (20x) |
| Resin-dentin bond stability of etch-and-rinse adhesive systems with different | Miranda et al (2020) | Quantitative (Energy Dispersive Analytical X-ray Spectometry ) | Human Teeth | Not mention | 50% ammoniacal AgNO3 in the dark for 24h + photodevoloping solution for 8h under fluorescent light + SEM + Energy-dispersive X-ray spectroscopy |

| | | | | | |
|---|---|---|---|---|---|
| concentrations of MMP inhibitor GM1489 | | | | | |
| New antimicrobial and collagen crosslinking formulated dentin adhesive with improved bond durability | Daood et al (2020) | Semi-quantitative | Human Teeth | Direct Class I | 50% ammoniacal $AgNO_3$ for 24h + photodevoloping solution under fluorescent ligh 8h + SEM |
| Validation of a method of quantifying 3D leakage in dental restorations | Rizzante et al (2020) | Quantitative (Micro-CT) | 3ºmolars | Direct Class II | 1. All specimens were scanned in a mCT device.<br>2. Subsequently, the specimens were immersed into 5 mL of 50% ammonium silver nitrate, for 12 hours in the absence of light.<br>3. Silver nitrate excess was removed with running water and wet cotton rolls, and a new mCT test scan (S2) was performed with the same parameters used for the baseline scan.<br>4. Scanned data were reconstructed using the mCT built-in software program.<br>5. Aligned images were exported to ImageJ.<br>6. Using the “Image Calculator” tool, aligned images were subtracted, showing only the silver nitrate |

| | | | | | |
|---|---|---|---|---|---|
| | | | | | infiltration around the restoration and specimen. |
| Comparative Evaluation of Efficacy of Four Different Materials in the Repair of Amalgam Restorations: Na in vitro Study | Chavan et al (2020) | Semi-quantitative (MSS) | premolars | Direct Class I | 1. 50% silver nitrate solution for 24 hours at 37°C + photodeveloping solution for 6 hours under continuous illumination to reduce the formation of precipitated silver ions<br>2. Section of the specimens + Stereomicroscope |
| Effect of different artificial aging protocols on marginal sealing ability of self-etch dental adhesives: micro-computed tomography evaluation | | Quantitative (Micro-CT) | 3ºmolars | Direct Class II | 50% ammoniacal $AgNO_3$ + Micro-CT for volumetric analysis |
| Evaluation of a Collagen-Reactive Monomer with Advanced Bonding Durability | Yu et al (2020) | Qualitative | 3ºmolars | Direct Class I | 50 wt% ammoniacal silver nitrate solution in the dark for 24 h, after which they were processed with a photo-developing solu- tion for 8 h and then further immersed in a fixing solution for 8 h. The specimens were rinsed with deionized water and sputter-coated with |

| | | | | | | |
|---|---|---|---|---|---|---|
| | | | | | | gold before being observed with a field emission scanning electron microscopy |
| | | | | | | |
| *Rhodamine-B* | Study on the Effect of Soft-Start Light on Microleakage in Pit and Fissure Closure | Chen et al (2023) | Semi-quantitative (MSS) | 3ºmolars | Direct Sealant Fissures | 0,1% a 24h + Posture microscope (40x) |
| | Comparative Evaluation of Microleakage of Bioactive, Ormocer, and Conventional GIC Restorative Materials in Primary Molars: Na In Vitro Study Microleakage of Three Restorative Materials. | Jain et al (2022) | Semi-quantitative (MSS) | Primary Molars | Direct Class V | 0,1% for 24h + Stereomiscoscope (40x) |
| | Effect of fluoride recharge on the microleakage of fluoride-releasing restorative materials: An ex vivo confocal laser scanning microscopy study | Gavini et al. (2022) | Semi-quantitative (MSS) | Human Teeth | Direct Class V | 0,5% for 10h + Confocal Laser Scanning Microscopy in flurescent mode |

| | | | | | |
|---|---|---|---|---|---|
| Effects of dimethyl sulfoxide pretreatment on the bonding properties of fluorotic dentin of different severity: an in vitro study | Zhang et al (2022) | Semi-quantitative (MSS) | Fluorotic Human Teeth | Enamel removal and restoration | 1% for 48h at 37º + Confocal Laser Scanning Microscopy in flurescent mode |
| Comparative Evaluation of Microleakage of Flowable Composite Resin Using Etch and Rinse, Self-Etch Adhesive Systems, and Self-Adhesive Flowable Composite Resin in Class V Cavities: Confocal Laser Microscopic Study | Sengar et al. (2022) | Semi-quantitative (MSS) | premolars | Direct Class V | 0,5% for 48h + stereomicroscope (10x) |
| Comparative Evaluation of Bond Strength and Microleakage of Three Ion-Releasing Restorative Materials at Various pH Levels | Kim et al. (2022) | Semi-quantitative (MSS) | premolars | Direct Class V | 0,02% for 24h + Confocal laser scanning Microscope |
| Effect of three different conditioning agents on cavosurface microleakage and | Khan et al (2022) | Semi-quantitative (MSS) | premolars | Direct Class V | ? for 24h + Stereomicroscope |

| | | | | | |
|---|---|---|---|---|---|
| bond strength of glass ionomer restorations - An in vitro study | | | | | |
| Comparing marginal microleakage in Class V cavities restored with flowable composite and Cention-N using confocal microscope- an in-vitro study. | Shenoi et al (2021) | Semi-quantitative (MSS) | premolars | Direct Class V | 0,1% for 48h + Confocal Laser sacning microscope |
| Effect of Enamel Pre-etching with Sodium Hypochlorite Deproteinisation and Bonding Agent on Retention and Microleakage of Pit and Fissure Sealants: An In-vitro Study | Mubeena et al (2021) | Semi-quantitative (MSS) | 3ºmolars | Direct Sealant Fissures | 0,2% for 24h + stereomicroscope (10x) |
| A Comparative Evaluation of Microleakage between Resin-Modified Glass Ionomer, Flowable Composite, and Cention-N in Class V Restorations: A Confocal Laser Scanning Microscope Study | Venugopal et al (2021) | Quantitative (ImageJ) | premolars | Direct Class V | 0,5% for 48h + Confocal Laser sacaning Microscopy (10x) |

| | | | | | |
|---|---|---|---|---|---|
| Effect of Different Application Techniques on Marginal Adaptation of Class II Cavities Filled with Three Different Bulkfill Composite Filling Material: An In Vitro Study using Confocal Microscopy | Albahari et al (2020) | Quantitative (ImageJ) | premolars | Direct Class II | ? + ? + Confocal Fluorescence Imaging Microscope (10x) |
| Evaluation of Microleakage in Cervically Placed Class Ii Restoration with an Alkasite Restorative Material and Bulk Fill Composite Resin Using Confocal Laser Scanning Microscope - An in-vitro Study | Aziz (2020) | Semi-quantitative (MSS) | Human Molars | Direct Class II | 0,5% for 24h + Confocal Laser sacanning Microscopy |
| Microleakage Assessment of Two Different Pit and Fissure Sealants: A Comparative Confocal Laser Scanning Microscopy Study. | Prabakar et al (2020) | Semi-quantitative (MSS) | 3ºmolars | Direct Sealant Fissures | 0,1% for 24h at 37ºC + termocycling + Confocal Laser sacanning Microscopy |
| | | | | | |

| | | | | | | |
|---|---|---|---|---|---|---|
| ***Toluidine*** | Effect of silver diamine fluoride on the microleakage of flowable resin composite and glass ionomer cement restorations to carious primary dentin: an- in vitro study | Osama et al (2024) | Semi-quantitative (MSS) | Primary Molars | Enamel removal and restoration | 1% por 14h + Stereomicroscope |
| | | | | | | |
| ***Thiazine*** | Microleakage Evaluation of Glass Hybrid Restoration Following Usage of Papain-Based Gel and Ceramic Bur for Caries Removal: An in. vitro Study | Alkhawaja et al (2022) | Quantitative (software Optika Vision lite 2.1) | Primary Molars | Carie excavation and restoration | 2% for 24h at 37ºC + Stereomicroscope (30x) |
| | | | | | | |
| ***India Ink*** | Effect of Different Cavity Disinfectants on Marginal Sealing Ability of a Seventh-generation Dentin Bonding Agent: An In Vitro Study. | Lokhande et al (2020) | Semi-quantitative (MSS) | Human Molars | Direct Class V | ? For 24h at 37ºC + Stereomicrocope (16x) |
| | | | | | | |

| | | | | | | |
|---|---|---|---|---|---|---|
| ***Imaging Techniques SEM*** | | | | | | |
| | Noninvasive assessment of novel nanohybrid resin cement adaptation using cross-polarization optical coherence tomography. | Naguib et al (2024) | Quantitative | Human Molars | Indirect | Specimens were sectioned and submitted to SEM assessmen |
| | Tooth-composite bond failure with a universal and an etch-and-rinse adhesive depending on mode and frequency of application | Schulz-Kornas et al (2024) | Qualitative | premolars | Direct Class V | Samples were dehydrated in an ascending alcohol series, dried with hexamethyldisilazane (HMDS), positioned on an SEM specimen holder, and sputter-coated with gold. Examination via SEM (500 to 3000 x) |
| | Structural and Mechanical Investigation of Class I Biomimetic Composite Dental Filling by X-ray Computed Tomography, Scanning Electron Microscopy, and Microtensile Bond Strength Testing | Borhy et al (2023) | Qualitative | Teeth | Direct Class I | gold spraying of the samples + SEM |
| | Comparative Evaluation of Microleakage in Hall's | Thakur et al (2023) | Qualitative | Primary Molars | Indirect | 1. Samples were cut into sections in a mesiodistal orientation along the tooth's longitudinal axis. |

| | | | | | |
|---|---|---|---|---|---|
| with SDF, Hall's, and Conventional Technique Using Different Luting Cements. | | | | | 2. All sections were ion sputtered with Au–Pd (gold palladium) by ion coating equipment + SEM |
| Stamp Technique: An Explorative SEM Analysis | Zotti et al (2023) | Quantitative (Imagej) | Human Teeth | Direct Class I | The samples were placed on an aluminium surface and metallised with a suitable metallising instrument. Evaluation by SEM |
| Evaluation of Marginal and Internal Fit of Ceramic Laminate Veneers Fabricated with Five Intraoral Scanners and Indirect Digitization | Al-Dwairi et al (2023) | Quantitative | Incisors | Indirect | SEM was used to examine sectioned specimens at a 200× magnification. |
| Effect of cervical margin relocation on marginal adaptation and microleakage of indirect ceramic restorations | Adel et al (2023) | Quantitative | 1ºMolars | Indirect | Specimens were examined for quantitative marginal analysis with SEM |
| Evaluating the effect of preheating on resin composites in pit-and-fissure caries treatments with a digital intraoral scanner | Yu et al (2023) | Quantitative | Human Molars | Indirect | Sample were cut in slice and placed in anhydrous ethanol and swished several times, and then stored in a drying oven at 37 °C for 24 h. + gold spraying + SEM |

| | | | | | |
|---|---|---|---|---|---|
| Microleakage, microgap, and shear bond strength of an infiltrant for pit and fissure sealing | Zhou et al (2023) | Qualitative | Human Molars | Direct Sealant Fissures | Specimens were gold-sputtered and inspected by SEM. |
| Comparative evaluation of microleakage of various restorative materials in pulpotomized primary molars | Thomas et al (2022) | Semi-quantitative (MSS) | Primary Molars | Indirect | specimens from each group were gold sputter-coated and examined under a SEM |
| Effect of Light-Curing Unit Type and Bulk-Fill Composite Resins with Different Photoinitiators on Marginal Gaps of Class II Restorations | Alavi et al (2022) | Quantitative (SEM software) | premolars | Direct Class II | Specimens gold-sputtering + SEM (2000x) |
| Microcomputed tomography evaluation of cement shrinkage under zirconia versus lithium disilicate veneers. | Hsu et al (2021) | Qualitative | Anterior Teeth | Indirect | The samples were then sputter coated with gold and observed under SEM |
| Sealing ability of new translucent zirconia crowns made with digital workflow and cemented with | Ferrari et al (2021) | Qualitative | Anterior Teeth | Indirect | Sample analysis was completed using a scanning electron microscope in at least one section from each sample at different magnifications. |

| | | | | | |
|---|---|---|---|---|---|
| different types of cement | | | | | |
| The effect of dentin age on the microshear bond strength and microleakage of glass-ionomer cements. | Techa-Ungkul et al (2021) | Qualitative | Human Molars | Direct Class V | The specimens were placed into an ultrasonic cleaner, air-dried and examined without gold sputtering using a SEM under low vacuum mode. Representative photomicrographs of the bonding interface were obtained at ×1500 magnification. |
| Comparison of three different bulk-filling techniques for restoring class II cavities: µCT, SEM-EDS combined analyses for margins and internal fit assessments | Putignano et al (2021) | Qualitative | Human Molars | Direct Class II | The specimens were fixed in metallic stubs and sputter-coated with gold + section of the samples + SEM |
| Microleakage Analysis of Different Bulk-Filling Techniques for Class II Restorations: µ-CT, SEM and EDS Evaluations. | Tosco et al (2020) | Qualitative | 3ºmolars | Direct Class II | The specimens were fixed in metallic stubs, sputter-coated with gold and observed by a scanning electron microscope (SEM). |
| External Marginal Gap Evaluation of Different Resin-filling Techniques for Class II Restorations-A Micro-CT and SEM Analysis | Sampaio et al (2020) | Qualitative | 3ºmolars | Direct Class II | SEM was performed for validation of the gaps and misfits observed in the lCT analysis. |

| | | | | | | |
|---|---|---|---|---|---|---|
| | Evaluation of Marginal Adaptation and Wear Resistance of Nanohybrid and Alkasite Restorative Resins | Afraaz et al (2020) | Quantitative | Human Molars | Direct Class II | Teeth samples were mounted on aluminium stubs, sputter-coated with gold, and assessed for quantitative marginal gaps under SEM |
| | | | | | | |
| ***Micro-CT*** | Structural and Mechanical Investigation of Class I Biomimetic Composite Dental Filling by X-ray Computed Tomography, Scanning Electron Microscopy, and Microtensile Bond Strength Testing | Borhy et al (2023) | Quantitative (VGSTUDIO MAX 3.4 software) | Human Teeth | Direct Class I | This study was performed using a dual-tube computed tomography equipment.The samples were reconstructed and evaluated using VGSTUDIO MAX 3.4 software. |
| | The Effect of Different Intermediary Layer Materials Under Resin Composite Restorations on Volumetric Cuspal Deflection, Gap Formation, and Fracture Strength | Deger *et al.* (2023) | Quantitative (Micro-CT) | premolars | Direct Class II | Each specimen was scanned twice with a micro-CT device.<br>1. The first time immediately after cavity preparation (T0 scan); the second time 24 hours after the restorative procedure (T1 scan). The T0 scan was used for superimposition with the other scan (T1) |

| | | | | | |
|---|---|---|---|---|---|
| Microcomputed tomography void analysis after cement cleanup methods | Peters *et al*. (2023) | Quantitative | Human Molars | Indirect | 1. All 20 specimens were scanned for marginal void analysis by using a mCT scanner.<br>2. Each specimen was analyzed by using an imaging processing software program. |
| X-ray microtomography analysis of gaps and voids in the restoration of non-carious cervical lesions with different composite resins. | Vieira *et al*. (2022) | Quantitative (Micro-CT) | premolars | Direct Class V | Each tooth was scanned twice using a μCT |
| The effect of composite placement technique on the internal adaptation, gap formation and microshear bond strength | Peskerso *et al*. (2022) | Quantitative (Micro-CT) | Human Molars | Direct Class V | Micro -CT + Image J Software |
| Microcomputed tomography evaluation of cement shrinkage under zirconia versus lithium disilicate veneers. | Hsu *et al*. (2021) | Quantitative (Amira Software) | Anterior Teeth | Indirect | The specimens were scanned Before light polymerization of the resin cements and after in the same settings in a dark room. After that, 2 specimens in each group were subjected to loading in a mastication simulator<br>2. The 3D image data were imported into Amira software to superimpose the scans and compare the cement layer before and after polymerization. |

| | | | | | |
|---|---|---|---|---|---|
| Comparison of three different bulk-filling techniques for restoring class II cavities: μCT, SEM-EDS combined analyses for margins and internal fit assessments | Putignano *et al.* (2021) | Quantitative (Micro-CT) | Human Molars | Direct Class II | 1. Each tooth was scanned by μCT. The total number of slices was approximately 950 for all the samples and a  a three- dimensional (3D) specimen reconstruction was generated. 2. The 3D rendering allowed void visualization within the intact restorative ma- terials and also provided quantitative measurements. 3. The 3D analysis software VG Studio MAX was used to perform image analysis and assessment of gap formation within the tooth-restoration interface. |
| Microleakage Analysis of Different Bulk-Filling Techniques for Class II Restorations: µ-CT, SEM and EDS Evaluations. | Tosco *et al.* (2020) | Qualitative (VGA Studio MAX ) | 3ºmolars | Direct Class II | The 3D analysis software VGA Studio MAX  was used to perform image analysis and assessment of gap formation within the tooth–restoration interface. |
| The use of a liner under different bulk-fill resin composites: 3D GAP formation analysis by x-ray microcomputed tomography. | Oglakci *et al.* (2020) | Quantitative (SkyScan) | premolars | Direct Class II | Gap formation analysis was performed with the microtomography system SkyScan |

| | | | | | | |
|---|---|---|---|---|---|---|
| | External Marginal Gap Evaluation of Different Resin-filling Techniques for Class II Restorations-A Micro-CT and SEM Analysis | Sampaio *et al.* (2020) | Quantitative ( ‘’3D measurement’’ of Micro-CT) | 3ºmolars | Direct Class II | For all groups, teeth were individually scanned before and after resin composite application + Both scans (empty and filled) were superimposed with the software Amira, and a subtraction was performed obtaining an image of the restoration only |
| | Volumetric change and gap formation in class V composite restorations: a micro-CT analysis | Oglakci *et al.* (2020) | Quantitative | Human Teeth | Direct Class V | 1. Each specimen was scanned four times with a micro-CT:(T0 scan): after cavity preparation; T1 scan (before light-curing); T2 scan (after light-curing); T3 scan (after thermomechanical aging). 2. After scanning, reconstruction was performed to obtain sectional images from projection images using NRecon ) software. |
| | | | | | | |
| | | | | | | |
| ***Stereomicroscope*** | Comparison of marginal accuracy in two different materials used in provisional crown and bridge - na in vitro experimental study | Javed *et al.* (2023) | Quantitative | Human Molar | Indirect | All four surfaces of the crown were observed under a stereomicroscope. |

| | | | | | | |
|---|---|---|---|---|---|---|
| | Marginal discrepancy and load to fracture of monolithic zirconia irconia laminate veneers: The effect of preparation design and sintering protocol | Saker *et al.* (2021) | Quantitative | Superior Incisors | Indirect | Marginal misfit was assessed using a stereomicroscope |
| | Influence of Bulk-fill Restoration on Polymerization Shrinkage Stress and Marginal Gap Formation in Class V Restorations | Correia *et al.* (2020) | Quantitative (AxioVision Software) | Bovine Incisors | Direct Class V | The marginal gap between the tooth structure and the restorative material was assessed along the margin of the restoration at a magnification of 50x using a stereomicroscope |
| | Influence of thermomechanical aging on marginal gap of CAD-CAM and conventional interim restorations | Angwarawong *et al*. (2020) | Qualitative | Human Molar | Indirect | The marginal gap was remeasured again with stereomicroscope. |
| | | | | | | |
| | | | | | | |
| ***Digital Microscope*** | Marginal Fit of Porcelain Laminate Veneer Materials under Thermocycling Condition: An In-Vitro Study | Hanoon *et al.* (2021) | Quantitative (ImageJ) | premolars | Indirect | Sectioning Samples (Venners) + Digital microscope |

| | | | | | | |
|---|---|---|---|---|---|---|
| | Evaluation of a novel instrument for placement of dental sealants. | Stewart *et al*. (2020) | Qualitative | Human Molars | Direct Sealant Fissures | Seccioning of the sample and observation through digital microscope to verify the marginal adaptation |
| | | | | | | |
| | | | | | | |
| ***OCT*** | Evaluation of gap formation for different adhesive agents in primary teeth with optical coherence tomography. | Sakaryali *et al.* (2024) | Quantitative (ImageJ) | Primary Molars | Direct Class V | 1. OCT to take images from all borders of the restorations. Adjustments made to capture appropriate images can be seen in pseudocolors and grey scale.<br>2. Obtained images were evaluated with Image J Software |
| | Noninvasive assessment of novel nanohybrid resin cement adaptation using cross-polarization optical coherence tomography. | Naguib *et al.* (2024) | Quantitative (ImageJ) | Human Molars | Indirect | A CP-OCT system was used to provide an image and to analyze the gap at the interface. |
| | Tooth-composite bond failure with a universal and an etch-and-rinse adhesive depending on mode and frequency of application | Schulz-Kornas *et al*. (2024) | Quantitative (ImageJ) | premolars | Direct Class V | Twenty-five equidistant OCT B-scans per filling were extracted from the complete volume scan. The first and last OCT B-scans were defined by the start and end of the composite restoration. |
| | Role of non-carious cervical lesions multicausality in the | Ordonez *et al.* (2022) | Quantitative (ImageJ) | premolars | Direct Class V | SD-OCT + Image J |

| | | | | | | |
|---|---|---|---|---|---|---|
| | behavior of respective restorations | | | | | |
| *3D-LCM* | The Influence of Adhesive Strategy, Type of Dental Composite, and Polishing Time on Marginal Gap Formation in Class I-like Cavities | Barbosa *et al.* (2023) | Quantitative (3D-LCM) | Bovine Incisors | Direct Class I | The images were verified using two specific 3DCLM tools, a height filter that uses core contrast that allowed areas with gaps in the tooth-night composite interface to be identified and a linear marker that allowed the length of the gaps to be measured. . |
| *EDS* | Comparison of three different bulk-filling techniques for restoring class II cavities: µCT, SEM-EDS combined analyses for margins and internal fit assessments | Putignano *et al.* (2021) | Qualitative | Human Molars | Direct Class II | (EDS) were performed using the same samples, for validation of the gaps and misfits found out in the µCT reconstruction model.<br>1. the specimens were fixed in metallic stubs and sputter-coated with gold + section of the samples + the surfaces of the sectioned samples were finished and polished with a lapping machine usingsilicon carbide abrasive papers + EDS |

| | | | | | | |
|---|---|---|---|---|---|---|
| ***CLSM*** | Formulation and Characterization of Experimental Adhesive Systems Charged with Different Concentrations of Nanofillers: Physicomechanical Properties and Marginal Gap Formation | Correia *et al.* (2024) | Quantitative | 3ºmolars | Direct Class I | 1. The sum of gaps along the occlusal interface was measured with CLSM 2. Subsequenty a longitudinal cut in bucco-lingual direction was perfomed, so the mesiall adhesive interface was expose for analysis using CLSM. |
| ***Nuclear Medicine*** | Microleakage Study of a Bulk Fill over an Uncured Adhesive System | Neves *et. al.* (2022) | Quantitative | Human Molars and Pre-molars | Direct Class V | 1. Test group and positive control group were covered with two layers of nail varnish up to 2 mm from the margins around the restorations. For the other side, negative control group was covered by two layers of varnish over the entire surface, including the area of the restored cavity. 2. Immersion of all samples in a solution of sodium pertechnetate (99mTc- NaO4) solution for 3 h and then the varnish was completely removed. |

| | | | | | |
|---|---|---|---|---|---|
| Evaluation of Microleakage of a New Bioactive Material for Restoration of Posterior Teeth: An In Vitro Radioactive Model | Pinto *et. al.* (2023) | Quantitative | Human Molars and Pre-molars | Direct Class V | 1. Test group and positive control group were covered with two layers of nail varnish up to 2 mm from the margins around the restorations. For the other side, negative control group was covered by two layers of varnish over the entire surface, including the area of the restored cavity.<br>2. Immersion of all samples in a solution of sodium pertechnetate (99mTc- NaO4) solution for 3 h and then the varnish was completely removed. |
| | | | | | |

| | | | | | | |
|---|---|---|---|---|---|---|
| ***Microbiological Technique*** | Bacterial Colonization and Proliferation in Primary Molars following the Use of the Hall Technique: A Confocal Laser Scanning Microscopy Study | Elbahary *et. al.* (2023) | Quantitative | Primary Molars | Caries removal and restoration | 1. Each tooth was placed in a Eppendorf plastic tube and then inserted into a disposable glass scintillation vial. The system was then sterilized overnight using an ethylene oxide gas.<br>2. Simulation of Enterococcus faecalis Bacterial Infection: A growth medium for streptomycin-resistant T2-strain Enterococcus faecalis was prepared and autoclaved. To prevent contamination by additional bacterial species, 0.5 mg/mL streptomycin sulfate was added (since E. . faecalis is resistent). Each tooth specimen was filled from the coronal part of the root canal with the freshly prepared bacterial suspension and then incubated at 37 °C and 100% humidity. The bacterial suspension was replaced with a fresh preparation every 24 h for 21 days.<br>3. Preparation of samples for evaluation: After 21 days of incubation, the tooth specimens were embedded in a self-cure acrylic repair material, and mesiodistal coronal plane cuts were performed. The samples were stained using a LIVE/DEAD BacLight Bacterial Viability Kit L-7012 containing separate vials of the two-component dyes (SYTO 9 and propidium iodide in 1:1 mixture) for the staining of the biofilm. |

Immediately after the staining procedure, fluorescence from the stained bacteria was observed under a confocal laser scanning microscope.

- The extent of fluorescent staining within the evaluated areas was calculated.
- The distance between the bacterial load in the crown area and the pulp chamber was measured.
